# Achieving stable Na metal cycling via polydopamine/multilayer graphene coating of a polypropylene separator

**DOI:** 10.1038/s41467-021-26032-1

**Published:** 2021-10-01

**Authors:** Jieqiong Qin, Haodong Shi, Kai Huang, Pengfei Lu, Pengchao Wen, Feifei Xing, Bing Yang, Mao Ye, Yan Yu, Zhong-Shuai Wu

**Affiliations:** 1 State Key Laboratory of Catalysis, Dalian Institute of Chemical Physics, Chinese Academy of Sciences, 457 Zhongshan Road, 116023 Dalian, China; 2 College of Science, Henan Agricultural University, 63 Agricultural Road, 450002 Zhengzhou, China; 3 Dalian National Laboratory for Clean Energy, Dalian Institute of Chemical Physics, Chinese Academy of Sciences, 457 Zhongshan Road, 116023 Dalian, China; 4 University of Chinese Academy of Sciences, 19A Yuquan Road, 100049 Shijingshan District, Beijing, China; 5 Hefei National Laboratory for Physical Sciences at the Microscale, Department of Materials Science and Engineering, Key Laboratory of Materials for Energy Conversion, University of Science and Technology of China, 230026 Hefei, China

**Keywords:** Energy, Batteries, Batteries, Two-dimensional materials

## Abstract

Sodium metal batteries are considered one of the most promising low-cost high-energy-density electrochemical energy storage systems. However, the growth of unfavourable Na metal deposition and the limited cell cycle life hamper the application of this battery system at a large scale. Here, we propose the use of polypropylene separator coated with a composite material comprising polydopamine and multilayer graphene to tackle these issues. The oxygen- and nitrogen- containing moieties as well as the nano- and meso- porous network of the coating allow cycling of Na metal electrodes in symmetric cell configuration for over 2000 h with a stable 4 mV overpotential at 1 mA cm^−2^. When tested in full Na || Na_3_V_2_(PO_4_)_3_ coin cell, the coated separator enables the delivery of a stable capacity of about 100 mAh g^−1^ for 500 cycles (90% capacity retention) at a specific current of 235 mA g^−1^ and satisfactory rate capability performances (i.e., 75 mAh g^−1^ at 3.5 A g^−1^).

## Abstract (web summary)

The development of future Na metal batteries relies on the cycling stability of the metallic anode. Here, the authors propose a polypropylene separator functionalized with polydopamine and multilayer graphene to enable stable and prolonged Na metal cell cycling.

## Introduction

The rapid growth of portable electronics, electric vehicle, autonomous aircraft, and smart grid has intensively stimulated the urgent requirements for low-cost, high-energy-density batteries^1–4^. Due to high theoretical capacity (1166 mAh g^−1^), low redox potential (−2.714 V vs. standard hydrogen electrode), natural abundance and low price, metallic sodium (Na) has been regarded as a highly competitive anode for next-generation rechargeable battery^5–9^. Unfortunately, its high reactive activity, large volume change, unstable solid electrolyte interphase (SEI), and uncontrollable dendritic growth bring about low Coulombic efficiency, limited cyclability, and even safety risk for high-energy-density Na metal batteries, such as Na-S^10^ and Na-O_2_ batteries^11^, substantially inhibiting their actual applications^5,12–15^. To overcome the issues, various strategies, including tailoring electrolyte formulation (e.g., highly concentrated electrolyte, fluoroethylene carbonate additive)^16,17^, using solid-state electrolytes (gel polymer with boron nitride, Na_3_Zr_2_Si_2_PO_12_)^18,19^, creating artificial SEI (e.g., Al_2_O_3_, sodium benzenedithiolate, graphene)^20–22^, and designing nanostructured Na anodes (e.g., Na@O-functionalized carbon nanotube networks, Na@porous Al, Na@carbonized wood)^14,23,24^, have been developed to suppress the growth of Na dendrites and realize stable and safe Na metal anodes. Nevertheless, these designs usually reveal single chemical or physical function for regulating Na dendrites, and faced high processing cost and limited scalability. Besides, constructing functional separators is considered as a more reliable and cost-effective way to realize uniform Na deposition from chemical molecule and physical structure levels^25–27^.

From the viewpoint of molecular level, polymer brushes with abundant polar functional groups (e.g., C=O, –OH, –COOH, and –NH–) can enhance the electrolyte wettability, provide robust SEI interface, and thus easily homogenize the alkali-metal ion distribution and nucleation^21,28–32^. In particular, structural two-dimensional (s-2D) graphene-like polymer materials (e.g., poly(N-isopropylacrylamide), polyacrylamide grafted graphene oxide (GO), and polypyrrole-GO heterostructure) with sheet-like structure, high specific surface area (SSA), abundant surface chemistry and good mechanical flexibility, show tremendous advantage to regulate alkali-metal deposition and physically restrain dendrite puncture^22,28,29,33^. From the perspective of structure design, defective graphene and ordered mesoporous structure can serve as nanoporous buffer and ion channels to homogenize alkali-metal ion distribution and deposition^33^. Therefore, the reasonable construction of definable s-2D mesoporous functional polymer heterostructure for realizing stable, dendrite-free Na metal anodes from chemical molecule and physical structure levels is highly competitive yet remains elusive.

In this work, s-2D mesoporous polydopamine-graphene (mPG) heterostructures with definable pore diameter and sheet thickness are developed for stable, high-capacity Na metal anodes. Using free-standing s-2D substrate of GO and variable mesoporous template of SiO_2_ nanospheres, s-2D sandwich-like mPG heterostructures with adjustable pore sizes (7, 12, 22 nm), tailored sheet thickness (14, 20, 28 nm), and high SSA (144, 157, 114 m^2^ g^−1^) are successfully synthesized via GO based hard-template strategy. Owing to sodiophilic polydopamine surface, defective graphene layer, uniform mesoporous structure and high SSA, the s-2D mPG heterostructures are used as separator coating to endow Na metal anodes with Coulombic efficiency of >99.5%, cycling stability of ~2000 h at 1 mA cm^−2^ with 1 mAh cm^−2^, and rate performance of 25 mA cm^−2^ with 25 mAh cm^−2^. As a consequence, coupled with carbon-coated Na_3_V_2_(PO_4_)_3_ (NVP@C) cathode, the mPG-based Na || NVP@C full cells demonstrate stable cyclability with 90% capacity retention over 500 cycles and rate capability showing a capacity of 75 mAh g^−1^ at 30 C (1 C = 117.6 mA g^−1^).

## Results

### Synthesis and physicochemical characterizations of s-2D mPG heterostructures

**Fig. 1:**
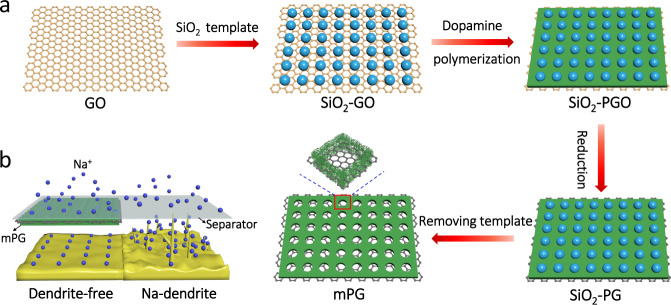
Scheme of s-2D mPG heterostructures for dendrite-free Na metal anode. **a** Schematic of the fabrication of s-2D mPG heterostructures, and **b** diagram of Na deposition behaviors with bare PP separator and mPG-coated PP separator.

The synthesis process of s-2D mPG heterostructures is schematically shown in Fig. 1a. First, the GO nanosheets modified by polydiallyldimethylammonium chloride are employed as s-2D free-standing substrates. Then, monodispersed SiO_2_ nanospheres with negative charge serve as mesoporous templates to orderly assemble on the surface of GO substrates through electrostatic adsorption. Afterwards, homogeneous polydopamine (Supplementary Fig. 1) layer is patterned on the surface of as-prepared SiO_2_-GO nanosheets to form SiO_2_-PGO nanosheets. Finally, s-2D mPG heterostructures with defined mesopore size and sheet thickness are generated after hydrothermal treatment (defined as SiO_2_-PG) and SiO_2_ etching. It is worth noting that this template strategy can efficiently realize the strong coupling of sodiophilic polydopamine, defective reduced GO (rGO, Supplementary Fig. 2) and adjustable mesopores, which is highly conducive to construct multifunctional ion redistributors for dendrite-free Na metal anodes (Fig. 1b), as discussed below.

**Fig. 2:**
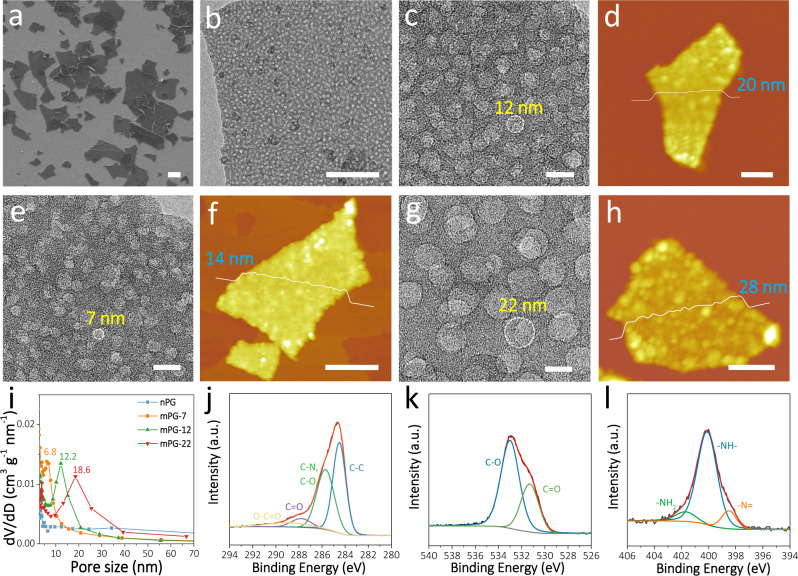
Characterization of s-2D mPG heterostructures. **a** SEM image, **b**, **c** TEM and HRTEM images, **d** AFM image and corresponding thickness analysis of mPG-12 nanosheets. **e** HRTEM image, **f** AFM image and corresponding thickness analysis of mPG-7 nanosheets. **g** HRTEM image, **h** AFM image and corresponding thickness analysis of mPG-22 nanosheets. **i** The pore size distribution curves of mPG-7, mPG-12, mPG-22, and nPG nanosheets. **j**–**l** High-resolution XPS (**j**) *C 1* *s*, (**k**) *O 1* *s*, and (**l**) *N 1* *s* spectra of mPG-12 nanosheets. Scale bars: **a** 1 μm, **b** 200 nm, **c**, **e**, **g** 20 nm, and **d**, **f**, **h** 500 nm.

The microstructure and chemical composition of s-2D mPG heterostructures are presented in Fig. 2. Taking mPG with 12 nm mesopores (mPG-12) nanosheets as example, scanning electron microscopy (SEM) image displays flat and uniform s-2D morphology with lateral size ranging from 0.5 to 3 µm (Fig. 2a). Transmission electron microscopy (TEM) and high-resolution TEM (HRTEM) images clearly indicate homogeneous mesoporous structure of mPG-12 nanosheets with uniformly ordered mesopores of ~12 nm (Fig. 2b, c). Atomic force microscopy (AFM) image reveals s-2D flat structure with a uniform thickness of ~20 nm (Fig. 2d). More importantly, the patterning of mesoporous polydopamine with definable pore size and thickness on graphene surface can be precisely controlled, e.g., by changing the size of SiO_2_ template (Fig. 2e–h and Supplementary Fig. 3). Specially, mPG-7 nanosheets show uniform mesopores of ~7 nm and an average thickness of ~14 nm, while mPG-22 nanosheets present ordered mesopores of ~22 nm and sheet thickness of ~28 nm. Meanwhile, nonmesoporous polydopamine-graphene (nPG) nanosheets were also synthesized (Supplementary Fig. 4) to highlight the importance of structure-directing SiO_2_ templates. Moreover, N_2_ adsorption and desorption isotherms of mPG-7, mPG-12, mPG-22, and nPG nanosheets represent type IV curves with H2-type hysteresis loop (Supplementary Fig. 5). Notably, mPG-12 nanosheets display larger SSA of 157 m^2^ g^−1^ than mPG-7 (144 m^2^ g^−1^), mPG-22 (114 m^2^ g^−1^), and nPG (54 m^2^ g^−1^) nanosheets (Supplementary Table 1). The dominated mesopore sizes of 6.8 nm for mPG-7, 12.2 nm for mPG-12, and 18.6 nm for mPG-22 nanosheets are well validated by the pore size distribution (Fig. 2i), nearly consistent with TEM observation. It is worth noting that the SSA generally changes with the variation of mesopore size and sheet thickness (Supplementary Figs. 5, 6). Fourier transform infrared spectroscopy (FT-IR) spectrum of mPG-12 reveals the existence of double peaks at 2930 and 2863 cm^−1^ featuring the indole structure, the characteristic vibration peak at 1712 cm^−1^ assigning to quinone (C=O) groups, and two distinct peaks at 1572 and 1462 cm^−1^ attributing to C=C resonance vibration and N-H bending vibration (Supplementary Fig. 7), demonstrative of the hybridization of polydopamine and graphene in mPG-12^30,34,35^. Furthermore, X-ray photoelectron spectroscopy (XPS) of mPG-12 confirms three strong signals of *C 1* *s*, *N 1* *s* and *O 1* *s* (Supplementary Fig. 8). The *C 1* *s* spectrum is fitted by four characteristic peaks of 284.5 ± 0.1 eV for CH_x_ and *sp*^2^-hybridized carbon, 285.7 ± 0.2 eV for C-O/C-N, 287.8 ± 0.2 eV for C=O, and 288.9 ± 0.2 eV for O-C=O (Fig. 2j)^34,36,37^. The *O 1* *s* spectrum exhibits two peaks centered at 533.0 ± 0.2 eV for O-C and 531.3 ± 0.1 eV for O=C (Fig. 2k)^34,36,37^. Moreover, the *N 1* *s* spectrum reveals three peaks at 401.7 ± 0.1 for –NH_2_, 400.0 ± 0.1 eV for –NH–, and 398.5 ± 0.1 eV for –N = functional group, respectively (Fig. 2l)^34,36,37^. It is evidenced that the mesoporous polydopamine layer is effectively grafted with graphene, and the abundant polar functional groups, e.g., C=O, –OH, and –NH–, are existed in s-2D mPG heterostructure.

### Computational investigation on the Na-ion affinity and redistribution effect of mPG

**Fig. 3:**
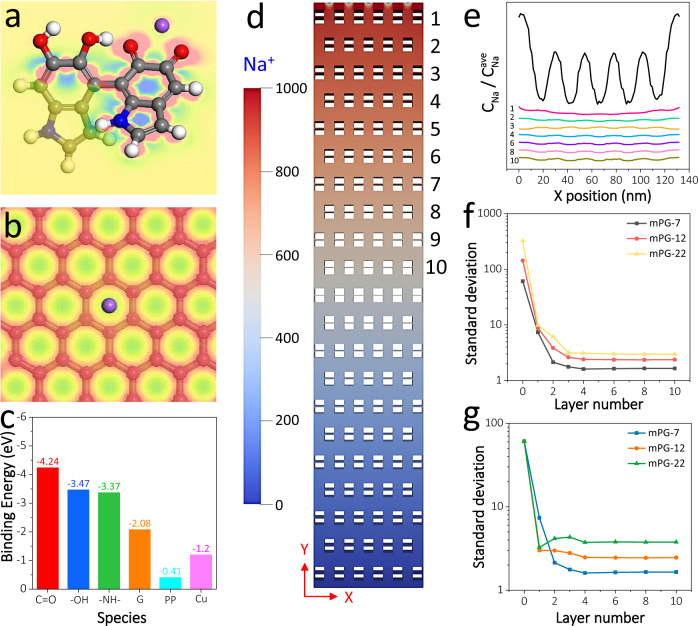
Sodiophilic nature and Na-ion deposition behavior of s-2D mPG heterostructures by DFT calculation and FVM simulation. **a** The deformation charge density of polydopamine molecule with Na. **b** The deformation charge density of graphene with Na. **c** Binding energies of Na with Cu, PP, graphene, and different functional groups in polydopamine. **d** Model diagram of Na ions through mPG layer (Inset: the color represents different concentration of Na ions). **e** The relative concentration of Na ions beneath different mPG layer with same amplitude and period of Na-ion distribution at the entrance. **f**, **g** Standard deviation of Na-ion concentration beneath mPG-7, mPG-12, and mPG-22 layers with (**f**) same amplitude and (**g**) same fluctuation of Na-ion distribution at the entrance.

To examine the effects of polar functional groups and s-2D mesoporous structure on Na metal deposition, density functional theory (DFT) calculation and finite volume method (FVM) simulation were conducted. Figure 3a-c and Supplementary Fig. 9 display the binding energies and charge densities between Na atom and polydopamine, graphene, PP and Cu. It can be seen that the interaction between Na and Cu (−1.20 eV) or PP (−0.41 eV) is much weaker than that between Na and polydopamine, owing to abundant polar groups (C=O of −4.24 eV, –OH of −3.47 eV, –NH– of −3.37 eV) in polydopmine. Thus, the sodiophilic nature of mPG coupled polydopamine and graphene with a high binding energy of −2.08 eV can serve as active site to realize more uniform ion transport and Na deposition. Furthermore, the effect of mesopore size of s-2D mPG on Na-ion distribution is investigated by FVM simulation. As shown in Fig. 3d and Supplementary Fig. 10, the migration of Na ions driven by electric field and diffusion flow through 20 layers of mPG nanosheets is taken into account for the simulation. Although mPG heterostructures are fragile pieces in reality, Na-ion flow driven by the vertical field between the two electrodes can be redistributed by s-2D mesoporous structure in mPG rather than perfect migration along the gaps between mPG nanosheets. As expected, with the shunting of mesopores in mPG, the distribution of Na ions becomes increasingly uniform as the ions migrate along the *Y*-axis, and achieves the steady stability after four layers of mPG (Fig. 3e and Supplementary Fig. 11). The mPG-7 reveals the lowest standard deviation whether with same amplitude and period or same fluctuation and period of Na-ion distribution at the entrance, implying the smaller mesopores are more beneficial to the redistribution and uniformity of Na ions (Fig. 3f, g). Further, the tunable SSA, mesopore size and thickness of mPG nanosheets can effectively regulate the Na-ion diffusion across the separator and interface, and further influence the process of Na plating and SEI formation. As shown in the electrochemical impedance spectroscopy (EIS) plots (Supplementary Fig. 12), the mPG-12 based Na || Cu half cell presents lower interface resistance (1.3 Ω) and charge transfer resistance (11.9 Ω), as well as higher Na-ion diffusion coefficient (5.7 × 10^−12^ cm^2^ s^−1^), compared with those of mPG-7 (2.8 Ω, 22.0 Ω and 2.7 × 10^−13^ cm^2^ s^−1^) and mPG-22 (2.1 Ω, 25.0 Ω and 2.6 × 10^−12^ cm^2^ s^−1^), possibly owing to larger SSA, appropriate mesopore size and thickness for efficient and uniform Na flux and deposition as well as stable SEI formation^38–41^. Taken the above into consideration, s-2D mPG heterostructures (especially mPG-12) possess huge merits of serving as multifunctional separator coating to homogenize distribution of Na ions and prevent “tip effects” (alkali-metal ion easily deposited on the tips of the protuberances of Cu foils to produce dendrites owing to the increased electric and ionic fields) of Na deposition^33,39,42^.

### Electrochemical characterizations in Na || Cu and Na || Na cell configurations

**Fig. 4:**
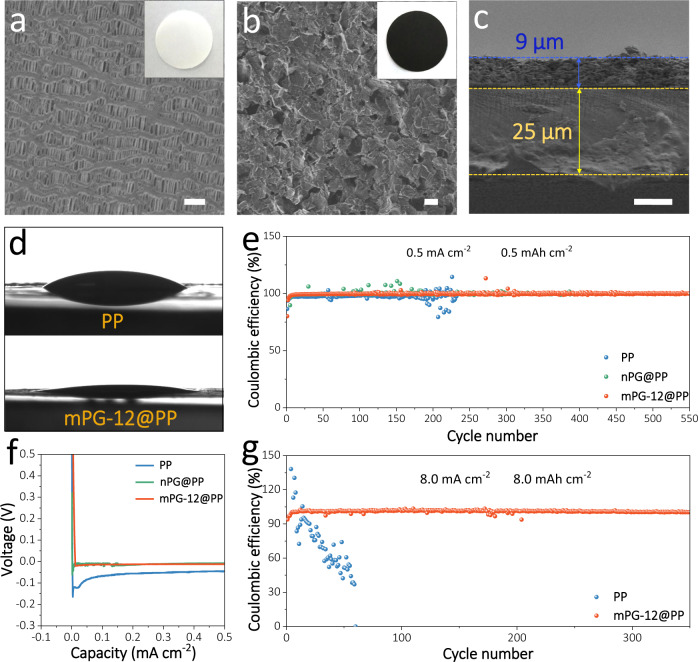
Characterization and performance of mPG-12@PP separator for Na || Cu cells. **a** Top-view SEM image and photograph (inset) of bare PP separator. **b** Top-view SEM image and photograph (inset) of mPG-12@PP separator. **c** Cross-section SEM image of mPG-12@PP separator. **d** Contact angles of the electrolyte on bare PP (top) and mPG-12@PP (bottom) separators. **e** Coulombic efficiencies of Na || Cu cells with mPG-12@PP, nPG@PP and PP separators tested at 0.5 mA cm^–2^, 0.5 mAh cm^–2^. **f** Voltage-capacity curves during Na nucleation with different separators. **g** Coulombic efficiencies of Na || Cu cells with mPG-12@PP and PP separators measured at 8.0 mA cm^–2^, 8.0 mAh cm^–2^. Scale bars: **a**, **b** 1 μm, and **c** 10 μm.

Considering the advantages of s-2D mPG heterostructures, we fabricated mPG-12 coated PP (mPG-12@PP) hybrid separators for Na-ion storage in a nonaqueous cell using Na metal as negative electrode. Compared with bare PP separator with plenty of large pores (~500 nm) and 25 µm thickness, mPG-12@PP separators exhibit uniform, layer-stacked film (only ~9 µm thickness) of s-2D mPG-12 nanosheets on the surface of PP, while retain good flexibility and well-defined pores (Fig. 4a–c and Supplementary Figs. 13, 14). Further, the contact angle of electrolyte on mPG-12@PP separator is ~8° (Fig. 4d), which is much lower than that on PP separator (31°), indicative of enhanced affinity of mPG-12@PP with electrolyte that is beneficial for Na-ion diffusion^28,43^.

To highlight the importance of mesopores in mPG-12, nPG-coated PP (nPG@PP) separator was also assembled. The electrochemical performance of Na || Cu asymmetric cells with mPG-12@PP, nPG@PP, and PP separators was first evaluated to illustrate Na deposition behavior. At a current density of 0.5 mA cm^−2^ with a cycling capacity of 0.5 mAh cm^−2^, the Na || Cu cells with mPG-12@PP separator exhibit stable plating/stripping process for 550 cycles (~1100 h) with a steady Coulombic efficiency of ~99.8%, which is better than that of nPG@PP (400 cycles with ~99.5% Coulombic efficiency) and PP separators (200 cycles with <90.0% Coulombic efficiency) (Fig. 4e and Supplementary Fig. 15). It can be well explained by the enlarged plating-stripping curves (Fig. 4f), in which the Na || Cu cells with mPG-12@PP separator show the lowest nucleation overpotential of only 20 mV than those of Na || Cu cells with nPG@PP (60 mV) and PP (170 mV) separators. The reduced nucleation overpotential verifies the low interface resistance and enhanced Na-ion transport kinetics through mPG-12@PP separator. Even at higher current density and areal capacity (4.0 mA cm^−2^ and 4.0 mAh cm^−2^; 8.0 mA cm^−2^ and 8.0 mAh cm^−2^), the Coulombic efficiency and cycling performance of Na || Cu cells with mPG-12@PP separator (~99.9% for 450 cycles; ~99.7% for 350 cycles) still outperform PP separator (~78.5% for 90 cycles; ~51.7% for 50 cycles) (Fig. 4g and Supplementary Figs. 16, 17). These values are also higher than those of recently reported Na metal anodes stabilized by various strategies^14,44–53^, demonstrating the advantage of mPG-12@PP separator for protecting Na metal anodes (Supplementary Table 2). To visually understand the impact of mPG-12@PP separator on Na plating/stripping process, the morphologies of Na metal anodes were traced by SEM (Supplementary Fig. 18). For the cell with bare PP separator, the anode exhibits Na dendrites with loosely moss-like structure at a Na loading of 1.0 mAh cm^−2^. In a sharp contrast, mPG-12@PP separator endows Na metal anode with a rather uniform and smooth surface without obvious dendrites, and this morphology becomes denser upon an increased Na loading of 2.0 mAh cm^−2^. The distinctive morphological transition of Na metal anodes with different separators demonstrates the key role of mPG-12 on efficient regulation of the Na-ion distribution and Na deposition. Meanwhile, SEM images of two sides of mPG-12@PP separator after 30 cycles were provided to visualize their changes. Prominently, the mPG-12 side reveals well-maintained flake-like and porous morphology, and the PP side keeps uniform, large pores on the surface, demonstrative of good structure stability of our mPG-12@PP separator (Supplementary Fig. 19).

**Fig. 5:**
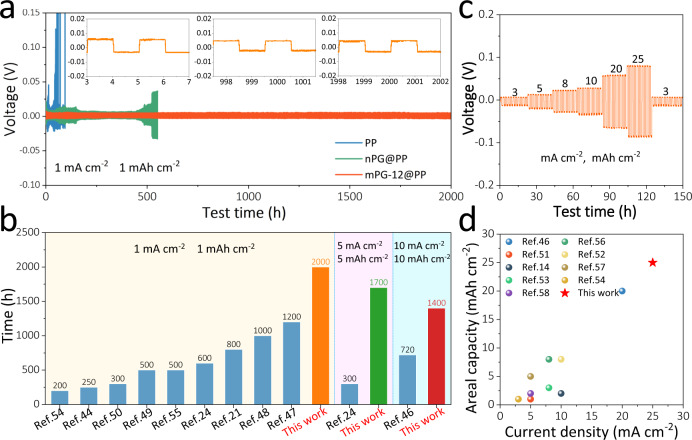
Electrochemical characterization of mPG-12@PP separator for Na || Na symmetric cells. **a** Voltage–time profiles of Na || Na cells with mPG-12@PP, nPG@PP, and PP separators at 1 mA cm^–2^, 1 mAh cm^–2^ (insets: magnified voltage profiles of Na || Na cell with mPG-12@PP at 5, 1000, and 2000 h, respectively). **b** Cycling stability comparison of Na || Na cells with mPG-12@PP separator and those of previously reported Na anodes stabilized by various methods at the same current densities and capacity. **c** Rate performance of Na || Na cells with mPG-12@PP separator obtained at different current densities and capacity, respectively. **d** Rate performance comparison of Na || Na cells with mPG-12@PP separator and other reported works recently.

Furthermore, symmetric Na || Na cells were fabricated to assess the voltage hysteresis and cycling stability. Figure 5a exhibits the voltage–time curves of Na || Na cells with mPG-12@PP, nPG@PP, and bare PP separators at 1 mA cm^−2^, 1 mAh cm^−2^. Notably, the cells with mPG-12@PP separator deliver virtually flat voltage plateaus over 2000 h with small overpotential (4 mV), illustrating their good interfacial stability and cyclability. In a sharp contrast, the cells with nPG@PP display higher voltage hysteresis after 500 h (>14 mV), and the cells with bare PP separators appear micro-short circuiting after dozens of hours (inset of Fig. 5a and Supplementary Fig. 20). When increasing the areal capacity to 5 mAh cm^−2^ or even 10 mAh cm^−2^, the cells with mPG-12@PP separator still show stable voltage plateaus over 1700 and 1400 h, respectively (Supplementary Figs. 21, 22). Remarkably, such a long cycle life of Na anodes (~2000 h at 1 mA cm^−2^ and 1 mAh cm^−2^, ~1700 h at 5 mA cm^−2^ and 5 mAh cm^−2^, and ~1400 h at 10 mA cm^−2^ and 10 mAh cm^−2^) is better than those of other Na anodes stabilized by various methods under similar test conditions, such as 3D MXene-melamine foam (720 h at 10 mA cm^−2^ and 10 mAh cm^−2^)^46^, poly(vinylidene difluoride)-coated Cu current collector (1200 h at 1 mA cm^−2^ and 1 mAh cm^−2^)^47^, and Na@rGO anodes (300 h at 5 mA cm^−2^ and 5 mAh cm^−2^)^24^, as shown as in Fig. 5b and Supplementary Table 3^21,44,48–50,54,55^. What’s more, the cells with mPG-12@PP separator reveal good rate capability under increasing current densities up to 25 mA cm^−2^ with 25 mAh cm^−2^ (overpotential ≈ 83 mV, Fig. 5c), whereas the voltage–time curve of the cells with PP separator suffers from severe fluctuation (Supplementary Fig. 23). To the best of our knowledge, such a high rate performance (25 mA cm^−2^, 25 mAh cm^−2^) greatly surpasses to those of the reported Na metal anodes stabilized by diversified strategies, such as 3D MXene-melamine foam (20 mA cm^−2^, 20 mAh cm^−2^)^46^, Na-Hg alloy (8 mA cm^−2^, 8 mAh cm^−2^)^56^, core-shell C@Sb nanoparticles (5 mA cm^−2^, 1 mAh cm^−2^)^51^, Sb_2_MoO_6_ microspheres (10 mA cm^−2^, 8 mAh cm^−2^)^52^, oxygen-functionalized carbon nanotube (10 mA cm^−2^, 2 mAh cm^−2^)^14^, rGO aerogel (5 mA cm^−2^, 5 mAh cm^−2^)^57^, Sn^2+^ pillared Ti_3_C_2_ MXene (8 mA cm^−2^, 3 mAh cm^−2^)^53^, porous Cu matrix (3 mA cm^−2^, 1 mAh cm^−2^)^54^, and 3D carbon felt (5 mA cm^−2^, 2 mAh cm^−2^)^58^ (Fig. 5d and Supplementary Table 4). Further, from the EIS test, symmetric Na || Na cells with mPG-12@PP separator exhibit smaller and more stable resistance than that with pure PP separator at different cycling stages (Supplementary Fig. 24). Consequently, the good cycling stability, high rate capability and small resistance variation suggest relatively rapid reaction kinetics and stable SEI layer of Na metal anodes with mPG-12@PP separator^28,33,59,60^.

### Electrochemical characterizations in Na || NVP@C cell configuration

**Fig. 6:**
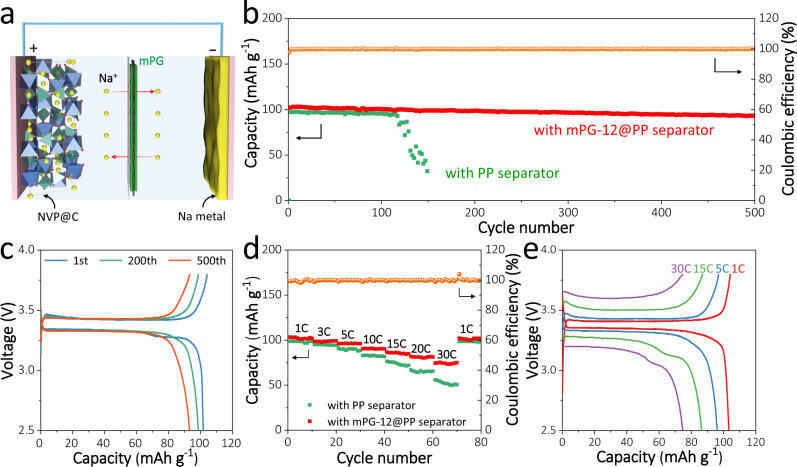
Electrochemical performance of Na || NVP@C full cells with mPG-12@PP and PP separators. **a** Schematic illustration of Na || NVP@C full cell with mPG-coated separators. **b** Cycling stability obtained at a current density of 2 C. **c** Galvanostatic charge-discharge profiles of Na || NVP@C full cells with mPG-12@PP separator at 2 C (1st, 200th, and 500th cycles). **d** Rate capability measured from 1 to 30 C. **e** Galvanostatic charge-discharge profiles of Na || NVP@C full cells with mPG-12@PP separator obtained at varying C-rate.

To highlight the applicability of mPG-coated separator, we investigated a full cell comprising of an as-deposited Na metal anode of 10 mAh cm^−2^, a NVP@C cathode active materials (Supplementary Fig. 25) and the mPG-coated separator (Fig. 6a)^5,61,62^. Figure 6b and Supplementary Fig. 26 exhibit the comparison of cycling stability of Na || NVP@C full cells with mPG-12@PP and PP separators at 2 C. Significantly, the Na || NVP@C cell with mPG-12@PP separator demonstrates stable cycling performance with 90% retention of the initial capacity over 500 cycles, which surpasses that of Na || NVP@C cell with bare PP separator (decaying rapidly after 120 cycles), and other reported NVP@C based Na batteries (Fig. 6b, c)^63,64^. Moreover, the full cell with mPG-12@PP separator reveals better rate capability than that with PP separator (Fig. 6d and Supplementary Fig. 27). Specifically, the discharge capacities of the full cell with mPG-12@PP separator are 103, 96, 86 and 75 mAh g^−1^ at 1, 5, 15, and 30 C, higher than those of cell with PP separator (99, 91, 76, and 55 mAh g^−1^; Fig. 6e and Supplementary Fig. 28). It is noted that the observed kink at high rates may be derived from the limited available sites for sodium insertion at the end of the discharge (Supplementary Fig. 29)^63,65,66^, low sodium diffusion coefficient at the end of discharge status (Supplementary Fig. 30)^41,67–69^, and relatively low ionic conductivity of the electrolyte of NaClO_4_ in propylene carbonate^65,70–74^. In addition, even at a reduced capacity of 5 mAh cm^−2^ for the as-deposited Na anode^48,50,53,60,75,76^, our Na || NVP@C full cell with mPG-12@PP separator still displays good cycling stability with ~93% retention over 200 cycles, further indicating the effectiveness of mPG-12 for stable and high-energy-density Na metal batteries (Supplementary Fig. 31). On the other hand, when the mass loading of NVP@C is increased from 1 to 5 mg cm^–2^, the Na || NVP@C full cells exhibit cycling stability of >92% retention over 100 cycles at 0.5 C (Supplementary Fig. 32). With the reduced deposition of Na anode (5 mAh cm^–2^) and relatively high loading of NVP@C (3.8 mg cm^–2^), our Na || NVP@C full cell with mPG-12@PP separator also reveals better rate performance (99 mAh g^–1^ at 1 C and 72 mAh g^–1^ at 20 C) compared with Na || NVP@C with PP separator (96 and 4 mAh g^–1^, Supplementary Fig. 33). Furthermore, mPG-12 coated separator also exhibits good performance for Cu || NVP@C cells (Supplementary Fig. 34). These results fully confirm that s-2D mPG-coated separator enable Na metal batteries with significantly enhanced Coulombic efficiency, cycling stability and rate performance.

## Discussion

The good performance of Na metal anodes is attributed to the reasonable design of s-2D multifunctional mPG heterostructure synergistically coupled the sodiophilic polydopamine surface, s-2D defective graphene and definable mesoporous structure. First, the polydopamine layer features abundant polar groups, e.g., C=O, –OH, and –NH–, endowing it with excellent electrolyte wettability and strong substrate adhesion to facilitate homogeneous Na-ion transport and suppress the growth of Na dendrites at molecular level. Second, s-2D rGO with intrinsic defects, atomic thickness and excellent flexibility, offers a homogeneous Na-ion delivery by this nanoporous buffer layer^25,33^. Third, the exposed mesoporous structure not only increases the accessible contact area between active polydopamine and electrolyte, but also provides definable Na-ion pathways to regulate local current density and terminate the “tip effect”. Consequently, s-2D mPG heterostructures as multifunctional separator coating substantially realize dendrite-free uniform Na deposition from chemical molecule to physical structure levels.

In summary, s-2D mPG heterostructures with definable pore size and sheet thickness have been demonstrated for stable and high-capacity Na anodes. Owing to the abundant sodiophilic groups, defective graphene and exposed mesoporous structure, s-2D mPG heterostructures endow uniform Na-ion transport and dendrite-free Na deposition, as confirmed by both theoretical and experimental analysis. Notably, the Na metal anodes with mPG-12@PP separator exhibit high Coulombic efficiency (>99.5%), good cycling stability (~2000 h), and landmark rate performance (25 mA cm^–2^ and 25 mAh cm^–2^). Further, s-2D mPG-coated separator realizes Na || NVP@C cells and Cu || NVP@C cells with improved electrochemical performance. Therefore, this strategy paves a new avenue for the design of s-2D mesoporous polymer materials towards next-generation safe, rechargeable sodium metal batteries.

## Methods

### Synthesis of GO

According to modified Hummers method^77,78^, 5 g of graphite powder was added to concentrated H_2_SO_4_ of 130 mL and stirred for 20 min in ice-water bath. Then, 2.5 g of NaNO_3_ was added with continue stirring for 2 h. Afterwards, 15 g of KMnO_4_ was slowly added to reaction solution within 1 h, and continued to stir for 2 h under ice-water bath. Further, the reaction solution was transferred to water bath of 35 °C. After reacting 1 h, 230 ml of high-purity water was slowly added with the temperature of <40 °C. Then, the reaction temperature was improved to 98 °C and kept for 30 min. When the reaction was finished, 400 ml of high-purity water and 10 ml of H_2_O_2_ were gradually added to stir for 1 h. Finally, brown GO suspension (~7 mg mL^–1^) was obtained by repeated centrifugation, dialysis, and ultrasonic dispersion.

### Synthesis of s-2D mPG heterostructures

Typically, 18 mg of GO was added into the polydiallyldimethylammonium chloride solution (0.2 wt%) of 75 mL and stirred for 24 h. Then, 100 mL monodisperse SiO_2_ nanospheres solution (1.5 wt%) was slowly added into the mixture under stirring for 15 h. After centrifugation to remove excess SiO_2_ nanospheres, the SiO_2_-GO nanosheets were collected. Afterwards, as-obtained SiO_2_-GO were dispersed in 150 mL water to pattern polydopmine layers by adding a certain amount of dopamine hydrochloride and 180 mg of Tris-base. Finally, s-2D mPG heterostructures were obtained after hydrothermal treatment at 180 °C for 12 h and etching SiO_2_ by HF solution. According to the sizes of SiO_2_ template with 7, 12, and 22 nm, and the addition amounts of dopamine hydrochloride for 450, 600, and 900 mg, the as-synthesized mPG heterostructures were defined as mPG-7, mPG-12, and mPG-22 nanosheets, respectively. For comparison, the nPG nanosheets without mesopores were also synthesized without using SiO_2_ template, while other steps kept same as mPG-12 nanosheets.

### Fabrication of functional composite separators

The mPG-12 and polyvinylidene difluoride (PVDF) binder with a mass ratio of 90:10 were mixed in N-methyl-2-pyrrolidinone (NMP, ~0.5 mL) with grinding ~30 min to form a viscous solution. Then, the as-prepared suspension was blade-coated onto one side of a commercial PP separator (Celgard 2400, ~25 µm) with a blade of 100 µm. After vacuum drying at 80 °C overnight and punching into disks with a diameter of 19 mm, the mPG-12@PP separator (~34 µm) was successfully obtained.

### Characterization

The morphology and composition of materials, separators, and electrodes were recorded by SEM (JSM-7800F), TEM (JEM-2100), AFM (Veeco nanoscope multimode II-D), N_2_ adsorption and desorption isotherm (Quadrasorb SI), FT-IR (Hyperion 3000), carbon/sulfur elemental analyzer (HORIBA EMIA-8100), XPS (ESCALAB 250Xi) equipped with monochromatic Al Kα source of 1486.6 eV, and contact angle meter (DSA100).

### Electrochemical measurement

All the cells were assembled with CR2016 coin cells in an Ar-filled glovebox (H_2_O < 1 ppm; O_2_ < 1 ppm), and all the electrochemical measurements were conducted using a LAND CT2001A battery system on a lab bench with the environmental temperature of ~26 °C. The electrolyte used for Na deposition was 1 M NaPF_6_ in diglyme (~40 µL), while the electrolyte applied to Na || NVP@C full cells was 1 M NaClO_4_ in propylene carbonate with 5 wt% fluoroethylene carbonates (~100 µL). The water content of these nonaqueous electrolytes is <20 ppm. The Na || Cu cells were assembled with Cu foil as the working electrode, Na disc (thickness: 500 µm; purity: 99.9%) as the counter/reference electrode, and the coated side of functional separators toward Cu foil. The Na || Cu cells were precycled five cycles from 0.1 to 3.0 V to stabilize the SEI, and then evaluated the Coulombic efficiency of Na plating/stripping. At the start of SoC (SoC=0, open-circuit voltage), EIS measurements were collected with an AC amplitude of 5 mV over a frequency range of 0.01 to 10^5^ Hz using a CHI 760E electrochemical workstation. The Na || Na symmetric cells were prepared with as-deposited Na–Cu foil as the working electrode, Na disc as the counter/reference electrode, and the coated side of functional separators toward the working electrode. The symmetric cells were applied to evaluate the long-time cycling stability of Na metal anodes with functional separators. The Na || NVP@C full cells were assembled with NVP@C based cathode (NVP@C mass loading of ~1.0 mg cm^–2^), as-deposited Na anode (10 mAh cm^–2^) and functional separators combined with glass fiber (~675 µm). Finally, Cu || NVP@C cells consisting of NVP@C based cathode (NVP@C mass loading of ~2.0 mg cm^–2^), Cu foil and functional separators were fabricated. Specifically, NVP@C was prepared by a sol-gel method^63,79^. 0.02 mol ammonium metavanadate was dissolved in 40 mL of deionized water under stirring at 80 °C for 1 h. Then, 0.03 mol ammonium dihydrogen phosphate and 0.01 mol sodium citrate dehydrate were added with stirring for 2 h. When increasing the temperature to 120 °C, the solution gradually became gel. After drying and grinding, the precursor was annealed at 350 °C for 4 h and then 800 °C for 8 h in argon atmosphere to achieve target NVP@C powder. Furthermore, the NVP@C based cathode was prepared through mixing NVP@C (~2.7 wt% of carbon coating verified by carbon/sulfur elemental analyzer)^41,80–83^, acetylene black and PVDF at a weight ratio of 80:10:10 in NMP (~0.3 mL) and then pasting on a carbon-coated Al current collector (~18 µm). The electrochemical performance of Na || NVP@C full cells and Cu || NVP@C cells were measured between 2.5 and 3.8 V at varying C-rate. For the galvanostatic intermittent titration (GITT) measurement, the Na || NVP@C cell was first charged/discharged at 0.5 C for five activation cycles. Then, the cell was charged and discharged at 0.5 C for 6 min, and relaxed for 60 min to reach the equilibrium state.

### Computational simulation

DFT calculation and FVM simulation were conducted to study sodiophilic nature and Na-ion transportation behaviors of s-2D mPG heterostructures. In the frame work of DFT, all the calculations were performed with Dmol^3^ code^13,14,49^. The generalized gradient approximation (GGA) combined with the Perdew-Burke-Enzerhof functional was employed to describe the exchange and correlation potential. The structural relaxations of polydopamine, graphene, PP, and Cu (111) models were carried out firstly, followed with the Na-adsorption simulation step. All the electrons were considered when tackling the electron-ion interactions for atoms in the whole system. For the convergence criteria, the SCF tolerance used was 1.0 × 10^–5^ Ha·atom^–1^, and the maximum force and displacement were set as 2.0 × 10^–5^ Ha·Å^–1^ and 5.0 × 10^–3^ Å, respectively. The binding energies of Na atom with different substrate models (C=O, –OH, –NH– groups of polydopamine, graphene, PP, and Cu (111) surface) were defined as the energy difference between the substrate model with Na atom (*E*_tot_) and the summation of Na atom (*E*_1_) and substrate system (*E*_2_): *E*_ads_ = *E*_1_ + *E*_2_ − *E*_tot_.

For FVM simulation, OpenFOAM software was used to investigate the effect of mesopore size on Na-ion distribution^84^. Two-dimensional simulation was conducted to study Na-ion concentration distribution through the different mPG layers. Migration of Na ions driven by electric field and diffusion flow was taken into account in the simulation, and the governing equation of Na-ion movement was shown below^85^:

1 $$\frac{\partial c}{\partial t}=-\nabla \cdot \overrightarrow{{{{{{\bf{N}}}}}}}$$

2 $$\overrightarrow{{{{{{\bf{N}}}}}}}=-D\nabla c+\mu c\overrightarrow{{{{{{\bf{E}}}}}}}$$

3 $$\overrightarrow{{{{{{\bf{E}}}}}}}=-\nabla \varphi$$

where *φ* is potential difference, $\overrightarrow{{{{{{\bf{E}}}}}}}$ is electric filed, *c* is Na-ion distribution, $\overrightarrow{{{{{{\bf{N}}}}}}}$ is flux of Na ions, *D* is diffusion coefficient, *µ* is ionic mobility. In the simulation, the effect of pore size on Na-ion distribution are compared. According to the SEM, TEM and AFM results, three different physical models with varying pore sizes were constructed. Figure 3d in the mansucript shows an example of the two-dimensional physical model. In the models, twenty crisscross layers based on the SEM result were constructed to simulate movement of Na ions. In each layer, six or five holes were constructed, and symmetry conditions were set to two sides to show the axial distribution of Na ions. Between the nearby layers was layer spacing, the *D* and *µ* were regarded as same as mPG layers, and the simulated number were set to 3.0 × 10^−10^ m^2^ s^–1^ and 3.0 × 10^–12^ m^2^ s^–1^ V^–1^. In order to simulate the homogeneous distribution property of mPG layer, similar cosine function distribution of Na ions as shown in Fig. 3e in the manuscript was set in the entrance of different physical models. The electric field was set uniform in the whole simulation regime, and the value was set to 0.02 V m^–1^ according to experimental result. The Na-ion concentration of bottom boundary (i.e., outlet) was set to 0. The inner boundaries induced by holes were set to zero gradient. Notably, two different simulations based on the varying entrance conditions were conducted. In one simulation, the entrance distribution of Na ions possessed same amplitude and period. In another simulation, the amplitude of entrance distribution of Na ions was adjusted to ensure same fluctuation.

## Supplementary information

Supplementary Information

## Data Availability

The experiment data that support the findings of this study are available from the corresponding authors upon reasonable requests.

## References

[1] Goodenough JB, Park KS (2013). The Li-ion rechargeable battery: a perspective. J. Am. Chem. Soc..

[2] Cheng XB, Zhang R, Zhao CZ, Zhang Q (2017). Toward safe lithium metal anode in rechargeable batteries: a review. Chem. Rev..

[3] Bruce, P. G., Freunberger, S. A., Hardwick, L. J. & Tarascon J.-M. Li-O_2_ and Li-S batteries with high energy storage. *Nat. Mater*. **11**, 19–29 (2012). 10.1038/nmat3191 22169914

[4] Ma L (2020). Dendrite-free lithium metal and sodium metal batteries. Energy Storage Mater..

[5] Wang Y (2019). Developments and perspectives on emerging high-energy-density sodium-metal batteries. Chem.

[6] Sun B (2020). Design strategies to enable the efficient use of sodium metal anodes in high-energy batteries. Adv. Mater..

[7] Lee B, Paek E, Mitlin D, Lee SW (2019). Sodium metal anodes: emerging solutions to dendrite growth. Chem. Rev..

[8] Fan L, Li X (2018). Recent advances in effective protection of sodium metal anode. Nano Energy.

[9] Vaalma C, Buchholz D, Weil M, Passerini S (2018). A cost and resource analysis of sodium-ion batteries. Nat. Rev. Mater..

[10] Wei S (2016). A stable room-temperature sodium-sulfur battery. Nat. Commun..

[11] Hartmann P (2013). A rechargeable room-temperature sodium superoxide (NaO_2_) battery. Nat. Mater..

[12] Xu X (2020). Quasi-solid-state dual-ion sodium metal batteries for low-cost energy storage. Chem.

[13] Wang H, Matios E, Luo J, Li W (2020). Combining theories and experiments to understand the sodium nucleation behavior towards safe sodium metal batteries. Chem. Soc. Rev..

[14] Ye L (2019). A sodiophilic interphase-mediated, dendrite-free anode with ultrahigh specific capacity for sodium-metal batteries. Angew. Chem. Int. Ed..

[15] Hu X (2017). Quasi-solid state rechargeable Na-CO_2_ batteries with reduced graphene oxide Na anodes. Sci. Adv..

[16] Lee J (2017). Ultraconcentrated sodium bis(fluorosulfonyl)imide-based electrolytes for high-performance sodium metal batteries. ACS Appl. Mater. Interfaces.

[17] Lee Y (2018). Fluoroethylene carbonate-based electrolyte with 1 M sodium bis(fluorosulfonyl)imide enables high-performance sodium metal electrodes. ACS Appl. Mater. Interfaces.

[18] Shim J (2017). 2D boron nitride nanoflakes as a multifunctional additive in gel polymer electrolytes for safe, long cycle life and high rate lithium metal batteries. Energy Environ. Sci..

[19] Zhou W, Li Y, Xin S, Goodenough JB (2017). Rechargeable sodium all-solid-state battery. ACS Cent. Sci..

[20] Luo W (2017). Ultrathin surface coating enables the stable sodium metal anode. Adv. Energy Mater..

[21] Zhu M (2020). Dendrite-free sodium metal anodes enabled by a sodium benzenedithiolate-rich protection layer. Angew. Chem. Int. Ed..

[22] Wang H, Wang C, Matios E, Li W (2017). Critical role of ultrathin graphene films with tunable thickness in enabling highly stable sodium metal anodes. Nano Lett..

[23] Liu S (2017). Porous Al current collector for dendrite-free Na metal anodes. Nano Lett..

[24] Wang A (2017). Processable and moldable sodium-metal anodes. Angew. Chem. Int. Ed..

[25] Foroozan T (2018). Synergistic effect of graphene oxide for impeding the dendritic plating of Li. Adv. Funct. Mater..

[26] Zhang C (2018). 2D materials for lithium/sodium metal anodes. Adv. Energy Mater..

[27] Tu NDK (2020). Co-solvent induced piezoelectric γ-phase nylon-11 separator for sodium metal battery. Nano Energy.

[28] Li C (2019). Two-dimensional molecular brush-functionalized porous bilayer composite separators toward ultrastable high-current density lithium metal anodes. Nat. Commun..

[29] Li N (2019). Normalized lithium growth from the nucleation stage for dendrite-free lithium metal anodes. Angew. Chem. Int. Ed..

[30] Kim PJ, Pol VG (2018). High performance lithium metal batteries enabled by surface tailoring of polypropylene separator with a polydopamine/graphene layer. Adv. Energy Mater..

[31] Ryou M-H (2012). Excellent cycle life of lithium-metal anodes in lithium-ion batteries with mussel-inspired polydopamine-coated separators. Adv. Energy Mater..

[32] Ryou MH, Lee YM, Park JK, Choi JW (2011). Mussel-inspired polydopamine-treated polyethylene separators for high-power Li-ion batteries. Adv. Mater..

[33] Shi HD (2020). A two-dimensional mesoporous polypyrrole-graphene oxide heterostructure as a dual-functional ion redistributor for dendrite-free lithium metal anodes. Angew. Chem. Int. Ed..

[34] Sun T (2016). A biodegradable polydopamine-derived electrode material for high-capacity and long-life lithium-ion and sodium-ion batteries. Angew. Chem. Int. Ed..

[35] Yue X, Liu H, Liu P (2019). Polymer grafted on carbon nanotubes as a flexible cathode for aqueous zinc ion batteries. Chem. Commun..

[36] Liu T (2017). Self-polymerized dopamine as an organic cathode for Li- and Na-ion batteries. Energy Environ. Sci..

[37] Liu T (2018). In situ polymerization of dopamine on graphene framework for charge storage applications. Small.

[38] Li W, Liu J, Zhao D (2016). Mesoporous materials for energy conversion and storage devices. Nat. Rev. Mater..

[39] Liu W, Lin D, Pei A, Cui Y (2016). Stabilizing lithium metal anodes by uniform Li-ion flux distribution in nanochannel confinement. J. Am. Chem. Soc..

[40] Bredar ARC, Chown AL, Burton AR, Farnum BH (2020). Electrochemical impedance spectroscopy of metal oxide electrodes for energy applications. ACS Appl. Energy Mater..

[41] Zhou Y (2020). A high-temperature Na-ion battery: boosting the rate capability and cycle life by structure engineering. Small.

[42] Li S (2018). Developing high-performance lithium metal anode in liquid electrolytes: challenges and progress. Adv. Mater..

[43] Liang J (2020). A nano-shield design for separators to resist dendrite formation in lithium-metal batteries. Angew. Chem. Int. Ed..

[44] Chu C (2019). Uniform nucleation of sodium in 3D carbon nanotube framework via oxygen doping for long-life and efficient Na metal anodes. Energy Storage Mater..

[45] Wang S (2020). Stable sodium metal batteries via manipulation of electrolyte solvation structure. Small Methods.

[46] Shi H (2020). 3D flexible, conductive, and recyclable Ti_3_C_2_T_x_ MXene-melamine foam for high-areal-capacity and long-lifetime alkali-metal anode. ACS Nano.

[47] Hou Z (2020). Poly(vinylidene difluoride) coating on Cu current collector for high-performance Na metal anode. Energy Storage Mater..

[48] Bao C (2020). Sodiophilic decoration of a three-dimensional conductive scaffold toward a stable Na metal anode. ACS Sustain. Chem. Eng..

[49] Sun B (2018). Dendrite-free sodium-metal anodes for high-energy sodium-metal batteries. Adv. Mater..

[50] Guo M (2020). Three dimensional frameworks of super ionic conductor for thermodynamically and dynamically favorable sodium metal anode. Nano Energy.

[51] Wang G (2020). Core-shell C@Sb nanoparticles as a nucleation layer for high-performance sodium metal anodes. Nano Lett..

[52] Lu X (2020). Enabling high-performance sodium metal anodes via a sodiophilic structure constructed by hierarchical Sb_2_MoO_6_ microspheres. Nano Energy.

[53] Luo J (2019). Pillared MXene with ultralarge interlayer spacing as a stable matrix for high performance sodium metal anodes. Adv. Funct. Mater..

[54] Wang C, Wang H, Matios E, Hu X, Li W (2018). A chemically engineered porous copper matrix with cylindrical core-shell skeleton as a stable host for metallic sodium anodes. Adv. Funct. Mater..

[55] Luo W (2017). Encapsulation of metallic Na in an electrically conductive host with porous channels as a highly stable Na metal anode. Nano Lett..

[56] Zhang Q (2020). A thermodynamically stable quasi-liquid interface for dendrite-free sodium metal anodes. J. Mater. Chem. A.

[57] Wu F (2019). Reduced graphene oxide aerogel as stable host for dendrite-free sodium metal anode. Energy Storage Mater..

[58] Chi S-S, Qi X-G, Hu Y-S, Fan L-Z (2018). 3D flexible carbon felt host for highly stable sodium metal anodes. Adv. Energy Mater..

[59] Zheng X (2020). Embedding a percolated dual-conductive skeleton with high sodiophilicity toward stable sodium metal anodes. Nano Energy.

[60] Li G (2018). Stable metal battery anodes enabled by polyethylenimine sponge hosts by way of electrokinetic effects. Nat. Energy.

[61] Huang CJ (2021). Decoupling the origins of irreversible coulombic efficiency in anode-free lithium metal batteries. Nat. Commun..

[62] Zhao Y, Adair KR, Sun X (2018). Recent developments and insights into the understanding of Na metal anodes for Na-metal batteries. Energy Environ. Sci..

[63] Zhang X (2019). Na_3_V_2_(PO_4_)_3_: An advanced cathode for sodium-ion batteries. Nanoscale.

[64] Guo D (2018). Achieving high mass loading of Na_3_V_2_(PO_4_)_3_@carbon on carbon cloth by constructing three-dimensional network between carbon fibers for ultralong cycle-life and ultrahigh rate sodium-ion batteries. Nano Energy.

[65] Chen S (2017). Challenges and perspectives for NASICON-type electrode materials for advanced sodium-ion batteries. Adv. Mater..

[66] Jian Z (2014). Atomic structure and kinetics of NASICON Na_x_V_2_(PO_4_)_3_ cathode for sodium-ion batteries. Adv. Funct. Mater..

[67] Zhang W (2020). Full activation of Mn^4+^/Mn^3+^ redox in Na_4_MnCr(PO_4_)_3_ as a high-voltage and high-rate cathode material for sodium-ion batteries. Small.

[68] Rui X (2020). A low-temperature sodium-ion full battery: superb kinetics and cycling stability. Adv. Funct. Mater..

[69] Zhang J (2020). A novel NASICON-type Na_4_MnCr(PO_4_)_3_ demonstrating the energy density record of phosphate cathodes for sodium-ion batteries. Adv. Mater..

[70] Ponrouch A (2015). Non-aqueous electrolytes for sodium-ion batteries. J. Mater. Chem. A.

[71] Sun Y (2020). Development and challenge of advanced nonaqueous sodium ion batteries. EnergyChem.

[72] Sun Y, Shi P, Xiang H, Liang X, Yu Y (2019). High-safety nonaqueous electrolytes and interphases for sodium-ion batteries. Small.

[73] Ponrouch A (2013). Towards high energy density sodium ion batteries through electrolyte optimization. Energy Environ. Sci..

[74] Ponrouch A, Marchante E, Courty M, Tarascon J-M, Palacín MR (2012). In search of an optimized electrolyte for Na-ion batteries. Energy Environ. Sci..

[75] Wu J (2020). Sodiophilically graded gold coating on carbon skeletons for highly stable sodium metal anodes. Small.

[76] Shi H (2019). Conducting and lithiophilic MXene/graphene framework for high-capacity, dendrite-free lithium-metal anodes. ACS Nano.

[77] Dreyer DR, Park S, Bielawski CW, Ruoff RS (2010). The chemistry of graphene oxide. Chem. Soc. Rev..

[78] Zhu Y (2010). Graphene and graphene oxide: synthesis, properties, and applications. Adv. Mater..

[79] Yao Y, Jiang Y, Yang H, Sun X, Yu Y (2017). Na_3_V_2_(PO_4_)_3_ coated by N-doped carbon from ionic liquid as cathode materials for high rate and long-life Na-ion batteries. Nanoscale.

[80] Liu T (2019). Sustainability-inspired cell design for a fully recyclable sodium ion battery. Nat. Commun..

[81] Yao Y (2020). Toward high energy density all solid-state sodium batteries with excellent flexibility. Adv. Energy Mater..

[82] Pi Y (2020). Methanol-derived high-performance Na_3_V_2_(PO_4_)_3_/C: from kilogram-scale synthesis to pouch cell safety detection. Nanoscale.

[83] Chen M (2020). Development and investigation of a NASICON-type high-voltage cathode material for high-power sodium-ion batteries. Angew. Chem. Int. Ed..

[84] Weller HG, Tabor G, Jasak H, Fureby C (1998). A tensorial approach to computational continuum mechanics using object-oriented techniques. Comput. Phy..

[85] Zhao CZ (2018). An ion redistributor for dendrite-free lithium metal anodes. Sci. Adv..

