# Supplementary material for: Achieving stable Na metal cycling via polydopamine/multilayer graphene coating of a polypropylene separator
Source: Nat Commun. 2021 Oct 1;12:5786. doi: 10.1038/s41467-021-26032-1 (PMC8486844; doi:10.1038/s41467-021-26032-1)
Supplement: Supplementary file 1 — Supplementary Information [file 41467_2021_26032_MOESM1_ESM.pdf]

Supplementary Information

Achieving stable Na metal cycling via polydopamine/multilayer graphene coating of a polypropylene separator

Qin et al.

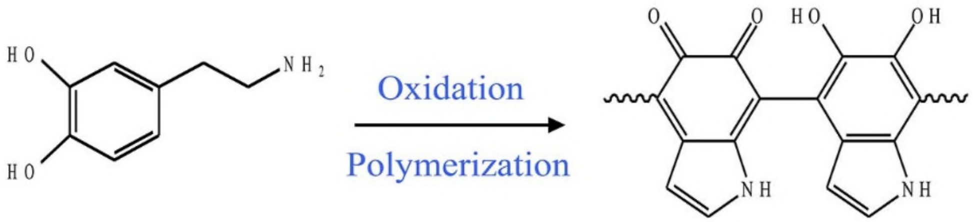

Supplementary Figure 1. **Synthesis and structure of polydopamine.**

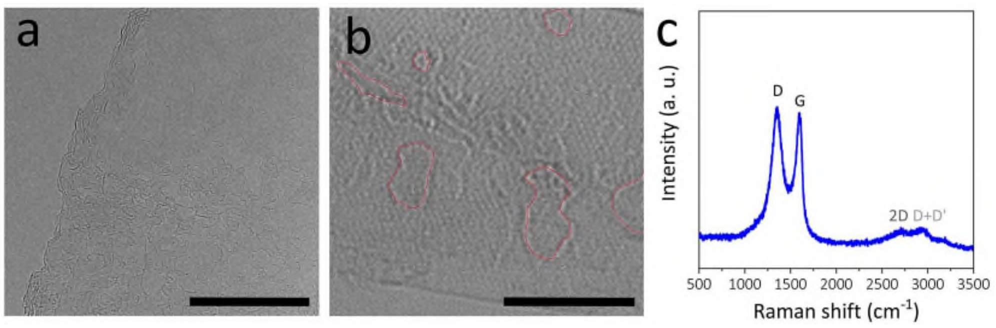

Supplementary Figure 2. Characterization of rGO nanosheets. (a, b) HRTEM images, showing a lot of edges, vacancies, nanopores and stacking faults. (c) Raman spectrum, in which the strong D peak, weak 2D peak and 1.04 of I_D/I_G ratio suggest relatively high state of defectiveness in rGO[1-4]. Scale bars: (a) 20 nm, and (b) 5 nm

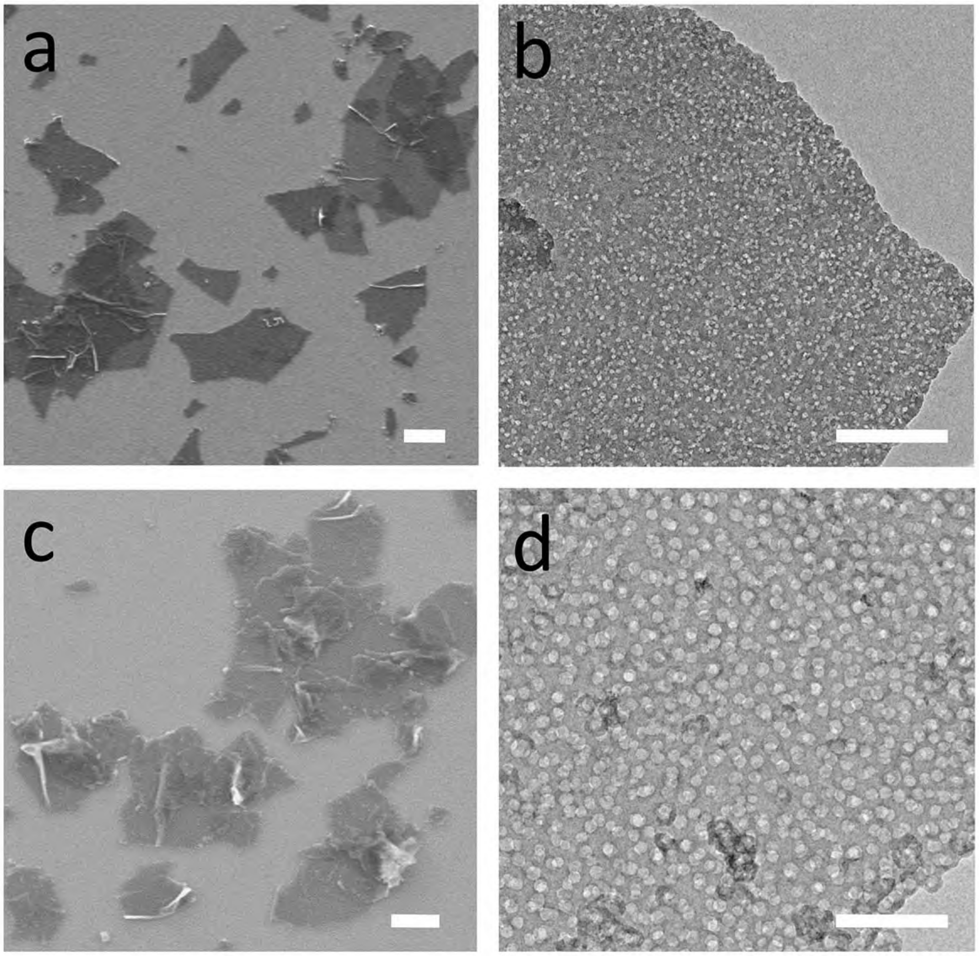

Supplementary Figure 3. Characterization of mPG-7 and mPG-22 nanosheets. (a) SEM image, and (b) TEM image of mPG-7 nanosheets. (c) SEM image, and (d) TEM image of mPG-22 nanosheets. Scale bars: (a, c) 1 μm, and (b, d) 200 nm

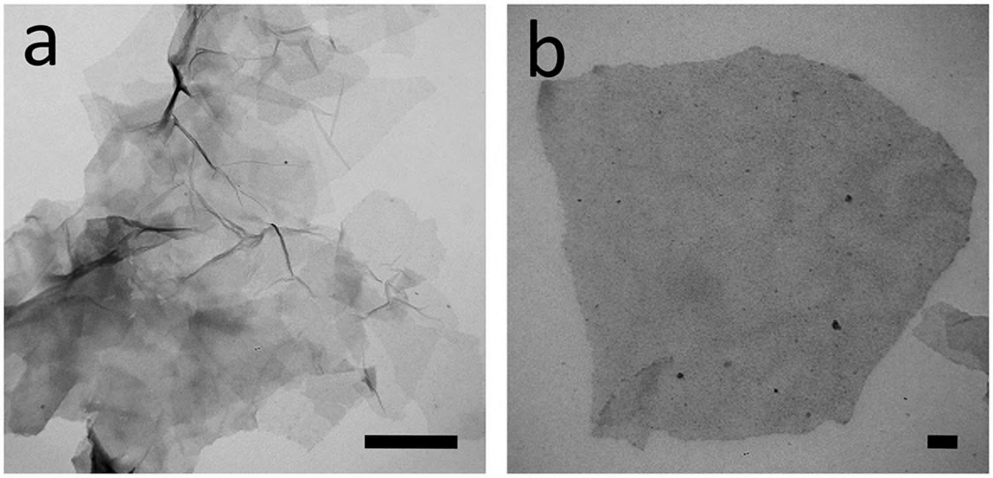

Supplementary Figure 4. TEM images of nPG nanosheets. Scale bars: (a) 500 nm, and (b) 100 nm

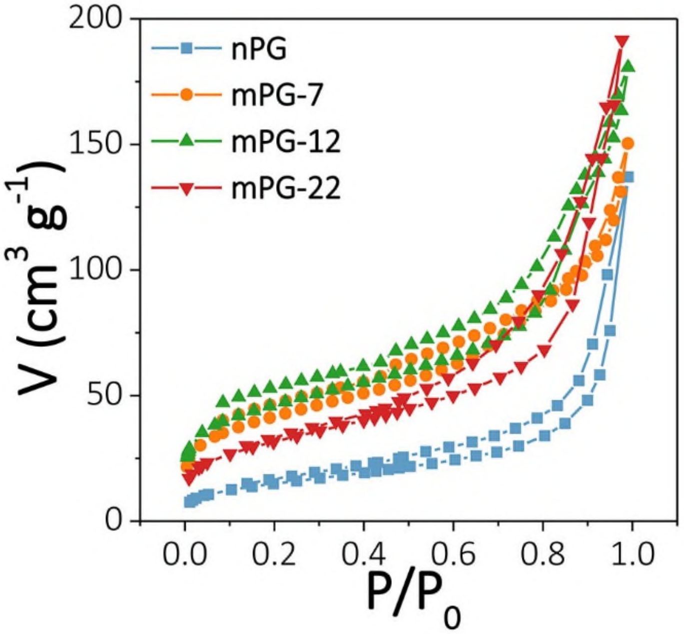

Supplementary Figure 5. N_2 adsorption and desorption isotherms of mPG-7, mPG-12, mPG-22 and nPG nanosheets, respectively.

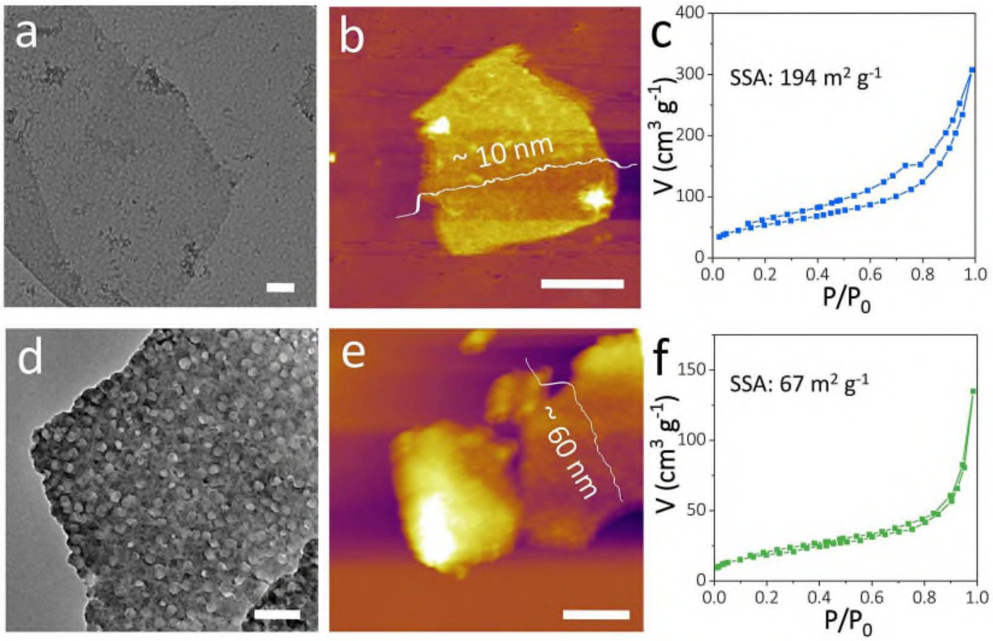

Supplementary Figure 6. Characterization of mPG-22 nanosheets with varying amount of dopamine precursor. (a) TEM image, (b) AFM image and corresponding thickness analysis, and (c) N_2 adsorption and desorption isotherm of mPG-22 nanosheets with 50% dopamine precursor. (d) TEM image, (e) AFM image and corresponding thickness analysis, and (f) N_2 adsorption and desorption isotherm of mPG-22 nanosheets with 200% dopamine precursor. Scale bars: (a, d) 100 nm, and (b, e) 500 nm

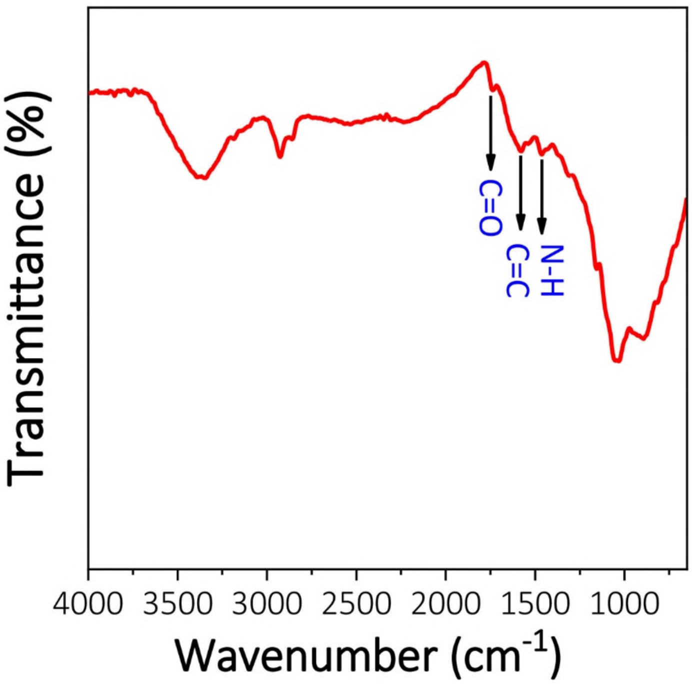

Supplementary Figure 7. FT-IR spectrum of mPG-12 nanosheets. The broad peak at 3300-3400 cm^{-1} is assigned to O-H and N-H stretching vibrations. The double peaks at 2930 and 2863 cm^{-1} feature the indole structure. The characteristic vibration peak at 1712 cm^{-1} is assigned to quinone (C=O) groups. And the two distinct peaks at 1572 and 1462 cm^{-1} are attributed to C=C resonance vibration and N-H bending vibration, respectively. The result demonstrates the hybridization of polydopamine and rGO, and the presence of substantial polar functional groups, including O-H, N-H, and C=O in mPG-12[5-8].

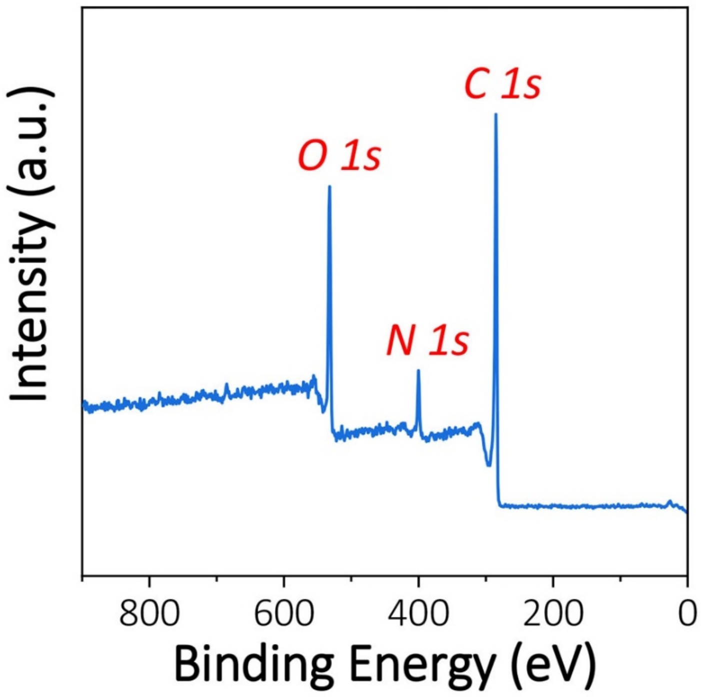

Supplementary Figure 8. XPS wide scan survey of mPG-12 nanosheets.

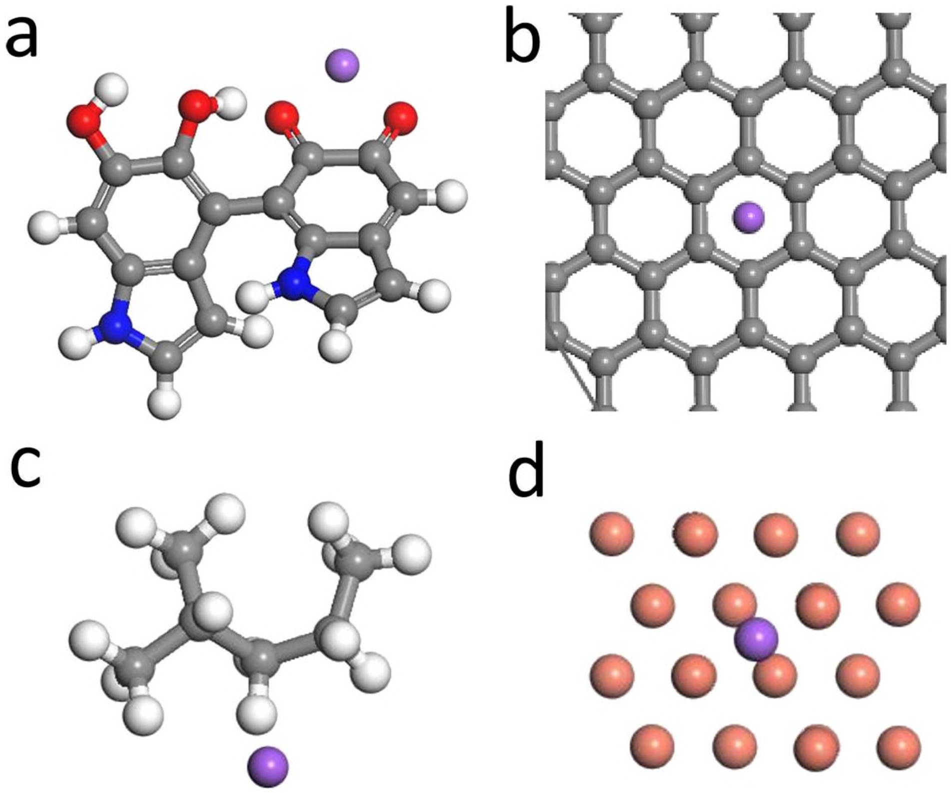

Supplementary Figure 9. Atomic models of (a) polydopamine, (b) graphene, (c) PP, and (d) Cu with Na atom used DFT calculation.

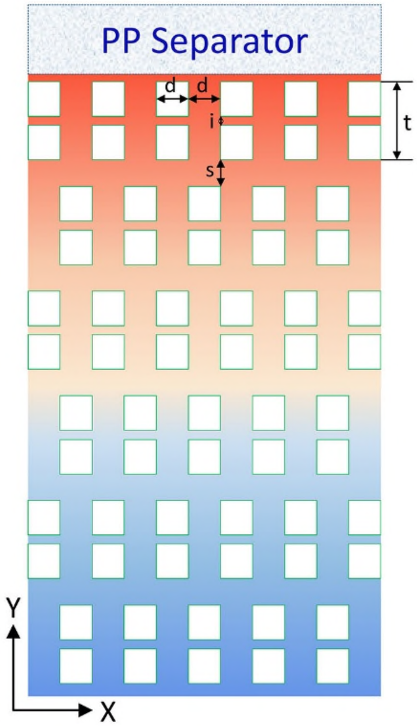

Sample	d (nm)	i (nm)	s (nm)	t (nm)
mPG-7	7	1	20	14
mPG-12	12	1	20	20
mPG-22	22	1	20	28

Supplementary Figure 10. FVM model parameters of Na ions through s-2D mPG-7, mPG-12 and mPG-22 layers, respectively.

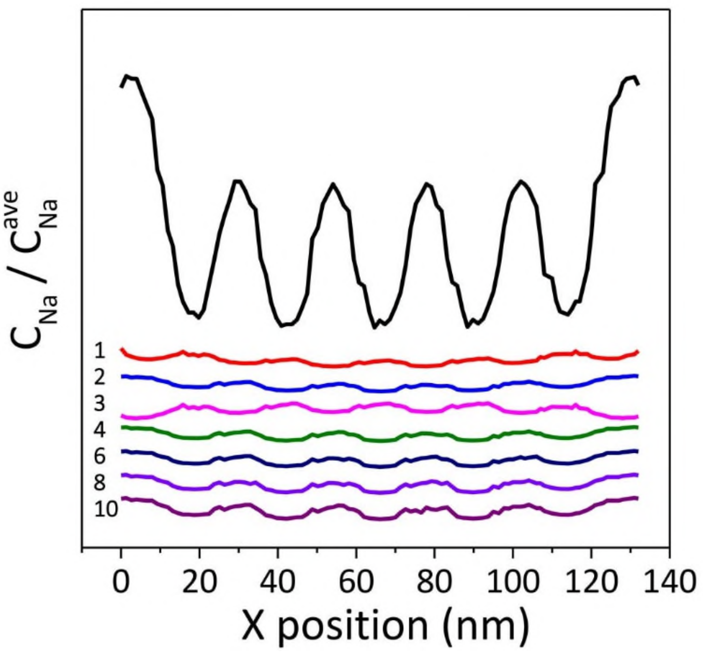

Supplementary Figure 11. The relative concentration of Na ions beneath different mPG layer with same fluctuation and period of Na ion distribution at the entrance.

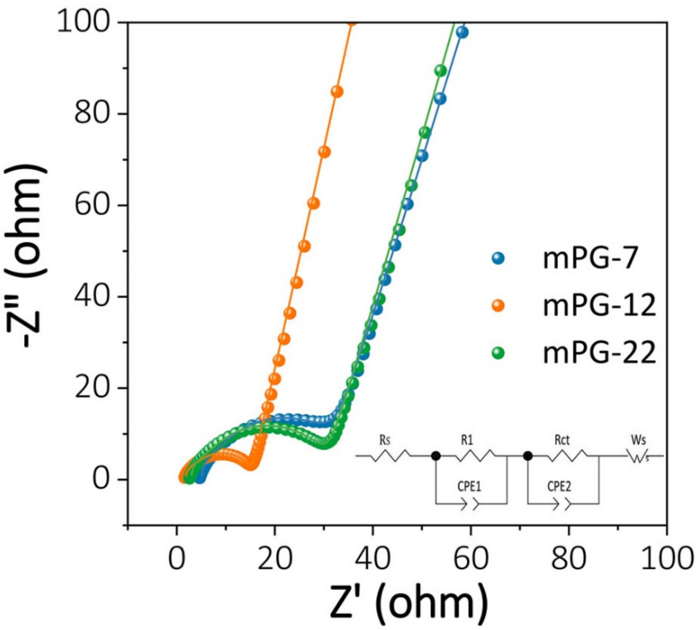

Supplementary Figure 12. EIS plots of Na || Cu cells with different mPG coated separators. The fitted lines are obtained using the inset equivalent circuit (Rs: equivalent series resistance, R_1: interface resistance, R_{ct}: charge transfer resistance, CPE_1, CPE_2: constant phase element, W_s: Warburg impedance)[9-12].

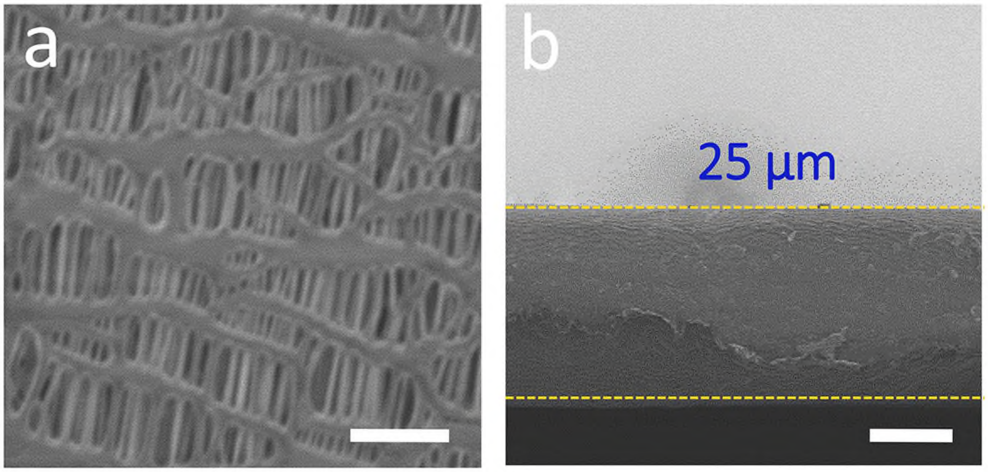

Supplementary Figure 13. Characterization of bare PP separator. (a) Top-view SEM image, and (b) cross-section SEM image of bare PP separator. Scale bars: (a) 1 µm, and (b) 10 µm

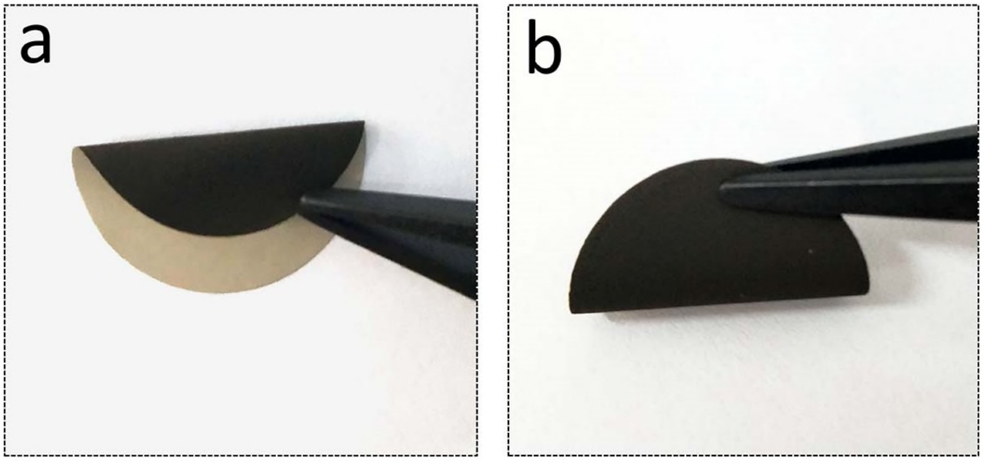

Supplementary Figure 14. (a, b) Digital photographs of mPG-12@PP separators with different bent states.

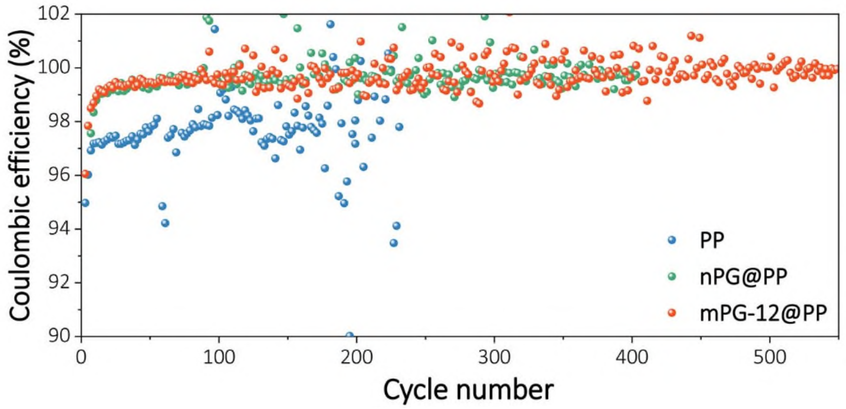

Supplementary Figure 15. Coulombic efficiency with a zoomed scale for Na || Cu cells with mPG-12@PP, nPG@PP and PP separators tested at 0.5 mA cm^{-2}, 0.5 mAh cm^{-2}.

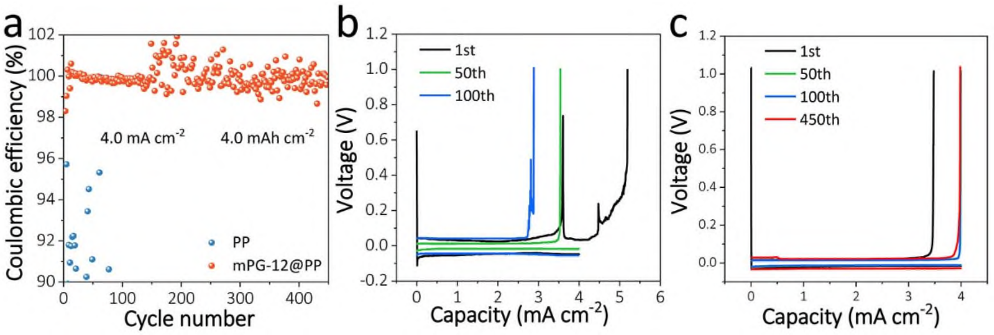

Supplementary Figure 16. Electrochemical performance of Na || Cu cells with PP and mPG-12@PP separators at 4.0 mA cm^{-2}, 4.0 mAh cm^{-2}. (a) Coulombic efficiency comparison. (b, c) Corresponding voltage profiles of Na || Cu cells with (b) PP, and (c) mPG-12@PP separator at different cycle.

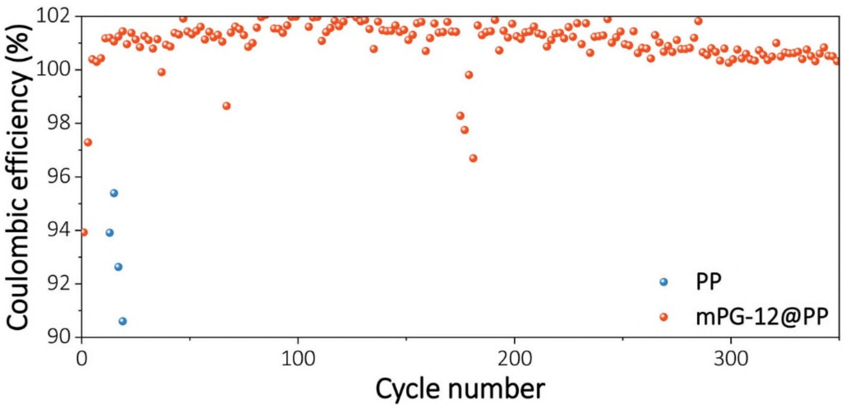

Supplementary Figure 17. Coulombic efficiency with a zoomed scale for Na || Cu cells with mPG-12@PP, nPG@PP and PP separators tested at 8.0 mA cm^{-2}, 8.0 mAh cm^{-2}.

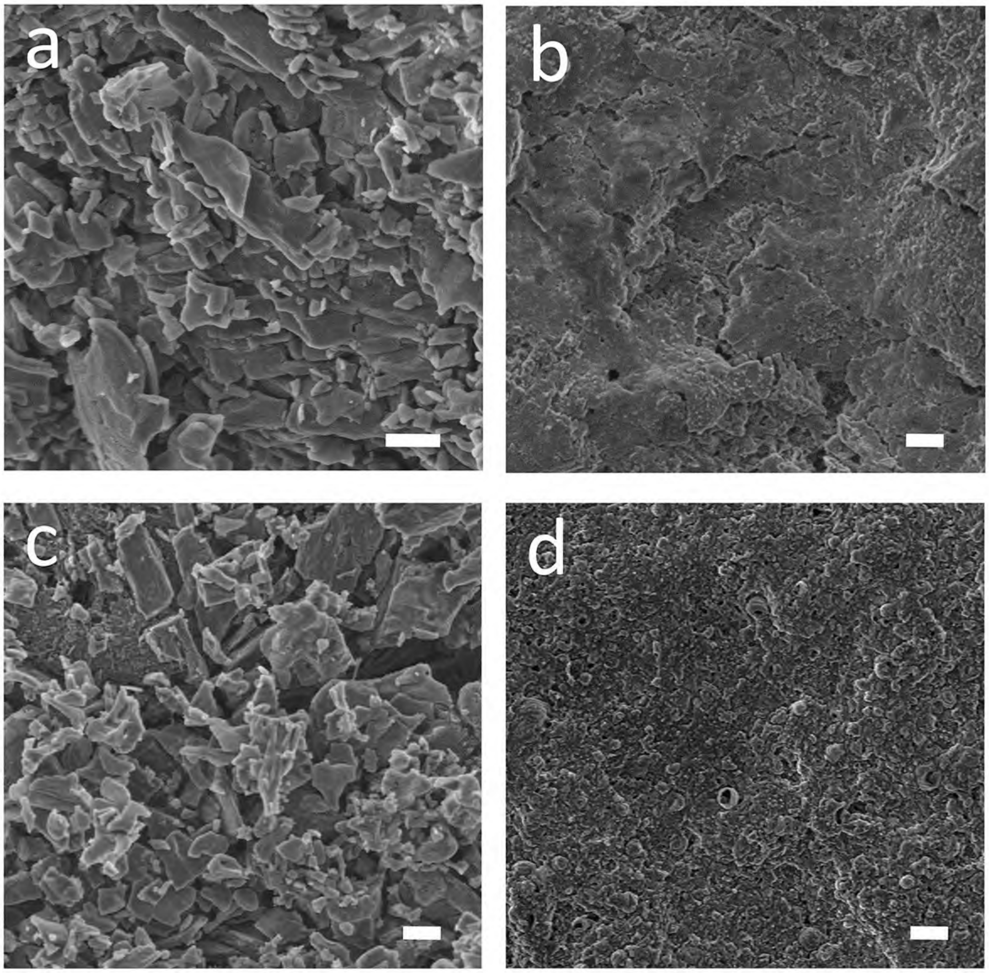

Supplementary Figure 18. Characterization of Na deposition with PP and mPG-12@PP separators. (a, b) Top-view SEM images of Na deposition with (a) PP, and (b) mPG-12@PP separators tested at 0.5 mA cm^{-2}, 1.0 mAh cm^{-2}. (c, d) Top-view SEM images of Na deposition with (c) PP, and (d) mPG-12@PP separators obtained at 0.5 mA cm^{-2}, 2.0 mAh cm^{-2}. Scale bars: 2 μm

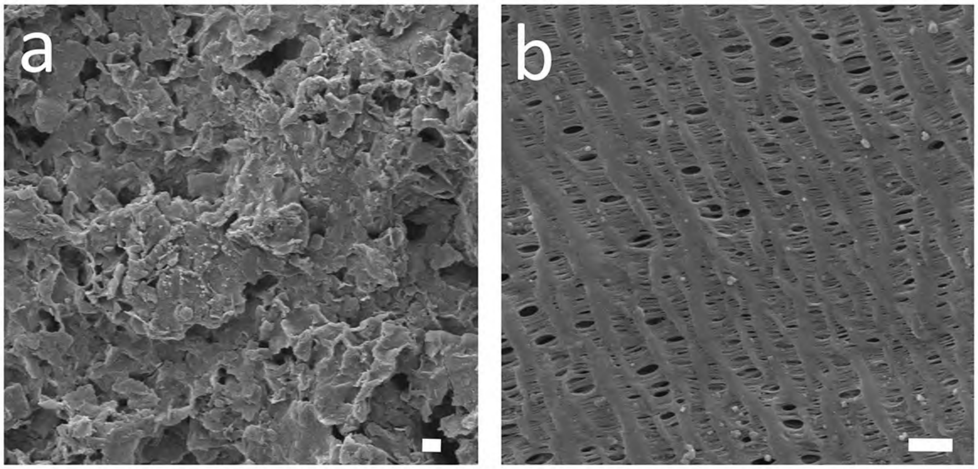

Supplementary Figure 19. SEM images of (a) mPG-12 side and (b) PP side of mPG-12@PP separator after several cycles. Scale bars: 1 μm

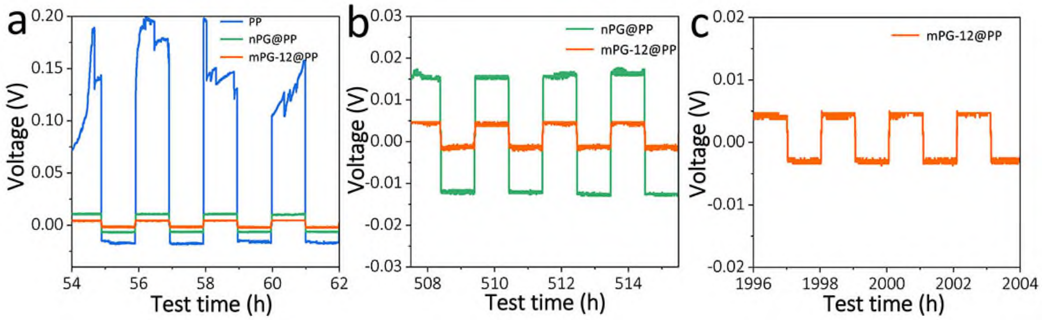

Supplementary Figure 20. Magnified voltage profiles of Na || Na cells with mPG-12@PP, nPG@PP and PP separators at different time. (a) ~60 h, (b) ~510 h, and (c) ~2000 h.

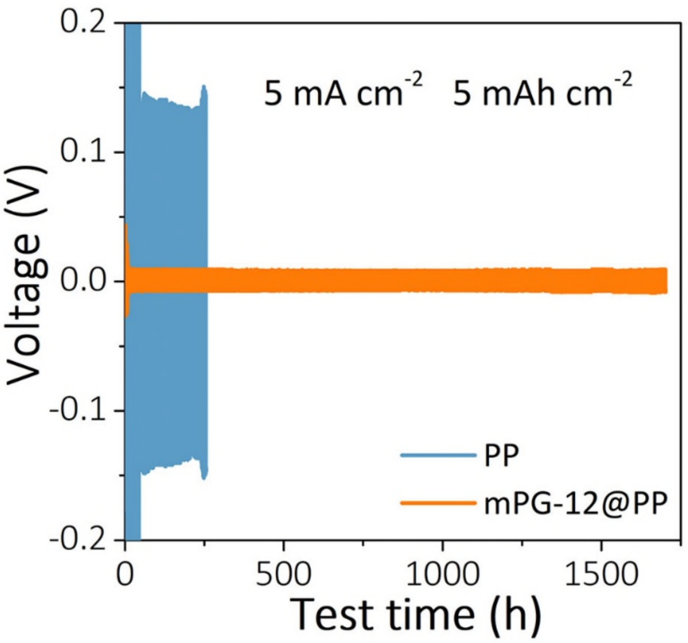

Supplementary Figure 21. Voltage-time profiles of Na || Na symmetric cells with mPG-12@PP and PP separators tested at 5 mA cm^{-2}, 5 mAh cm^{-2}.

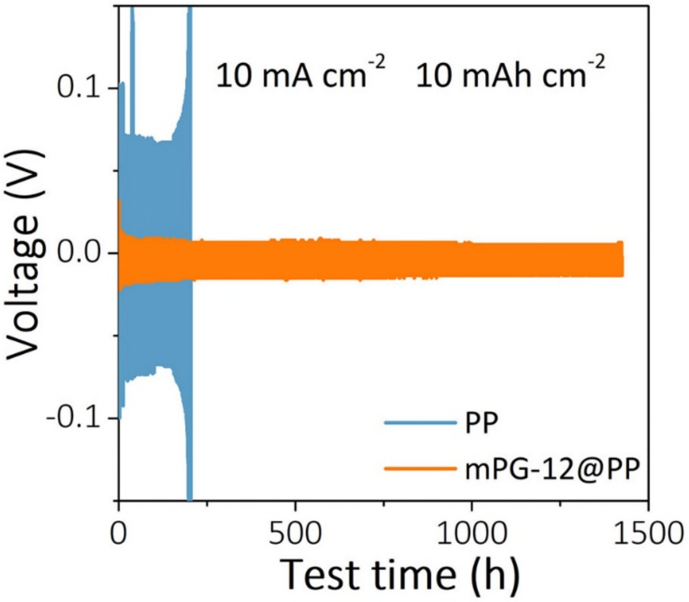

Supplementary Figure 22. Voltage-time profiles of Na || Na symmetric cells with mPG-12@PP and PP separators obtained at 10 mA cm^{-2}, 10 mAh cm^{-2}.

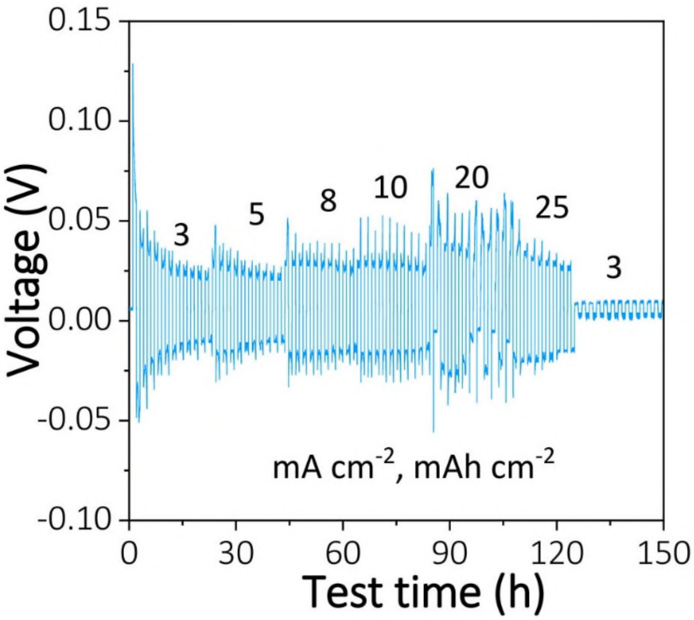

Supplementary Figure 23. Rate performance of Na || Na cells with PP separator at different current densities and capacity, respectively.

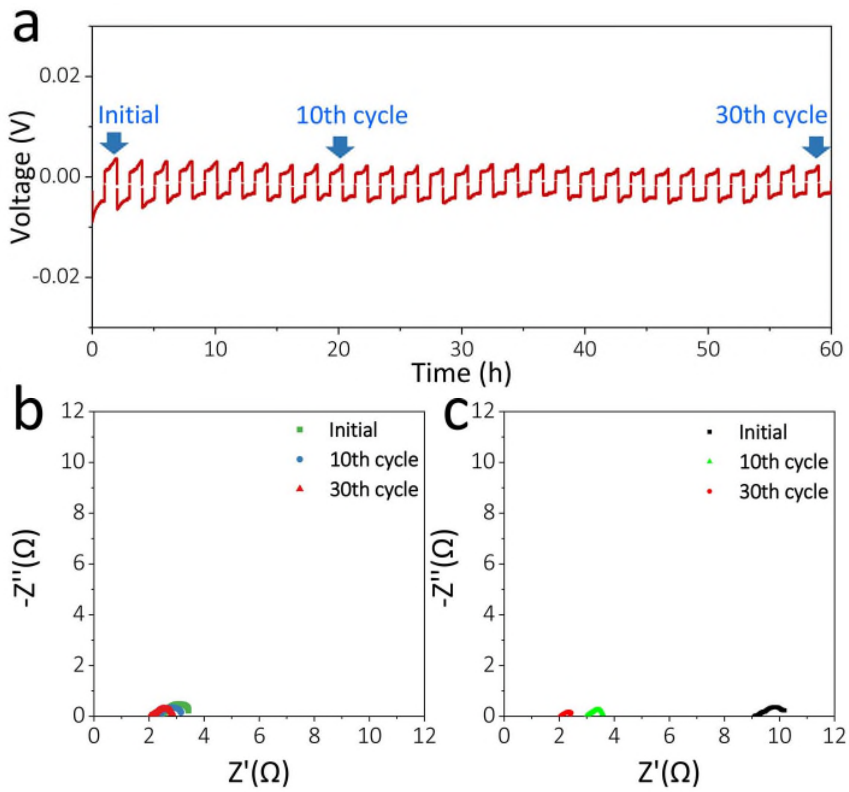

Supplementary Figure 24. (a) Voltage-time profile of Na || Na symmetric cells with mPG-12@PP separator at 1 mA cm^{-2}, 1 mAh cm^{-2}. (b, c) Nyquist plots of Na || Na symmetric cells with (b) mPG-12@PP and (c) PP separators at different cycling stages. Notably, the symmetric Na || Na cell with mPG-12@PP separator exhibits smaller and more stable internal impedance (intercept) and interfacial resistance (diameter of a semicircle) compared to that with PP separator. In good agreement with many related reports[11-15], the highly variable internal impedance and interfacial resistance of Na || Na cell with PP separator can be derived from uneven Na plating/stripping, uncontrollable dendritic growth, and continuously repaired SEI film. Therefore, our mPG-12@PP separator can guarantee more uniform Na deposition and more stable SEI layer, which have also been confirmed by good cycling stability and rate capability.

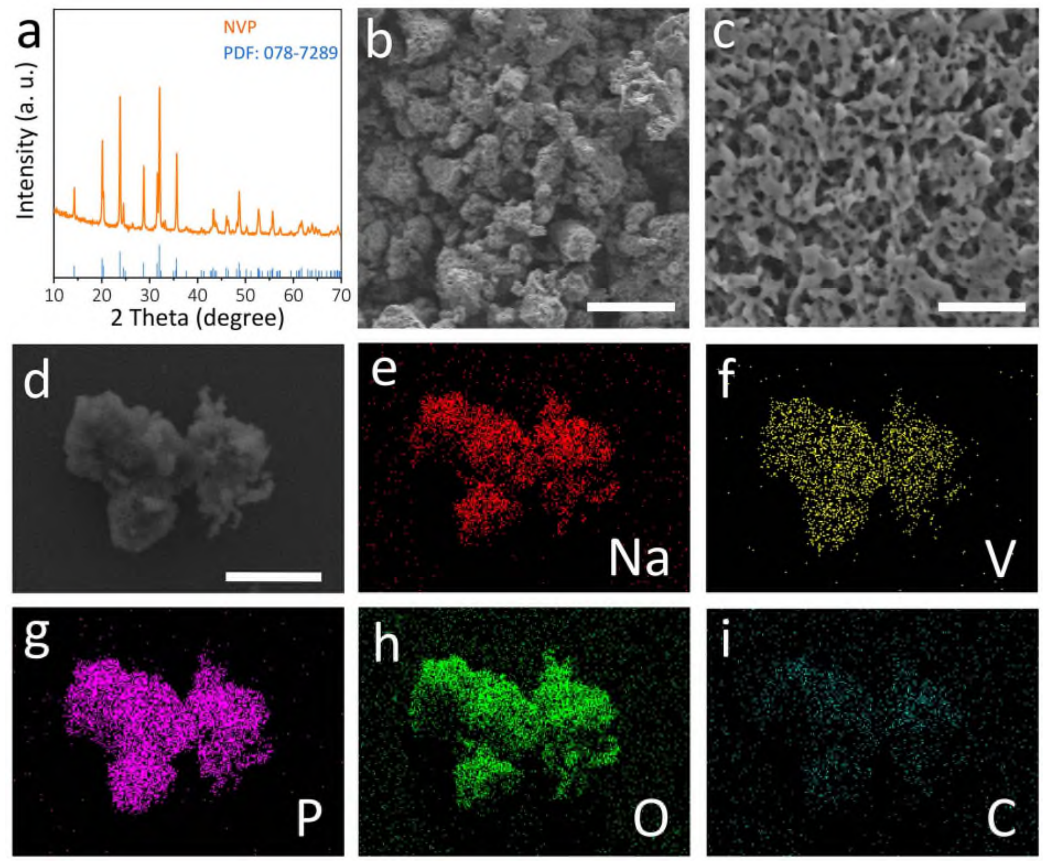

Supplementary Figure 25. Characterization of NVP@C. (a) XRD pattern, and (b, c) SEM images. (d) SEM image, (e) Na, (f) V, (g) P, (h) O and (i) C elemental mapping images of NVP@C. Scale bars: (b, d) 10 μm, and (c) 1 μm

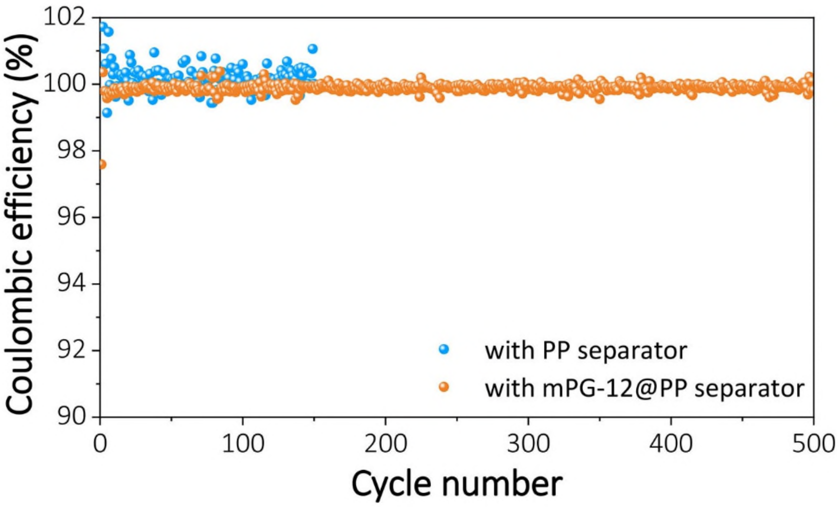

Supplementary Figure 26. Coulombic efficiency with a zoomed scale for Na || NVP@C full cells with mPG-12@PP and PP separators at 2 C.

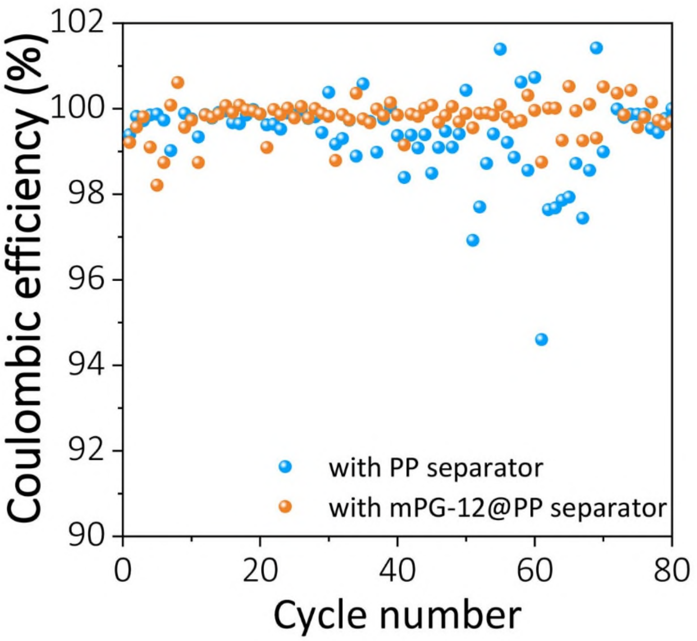

Supplementary Figure 27. Coulombic efficiency with a zoomed scale for Na || NVP@C full cells with mPG-12@PP and PP separators from 1 C to 30 C.

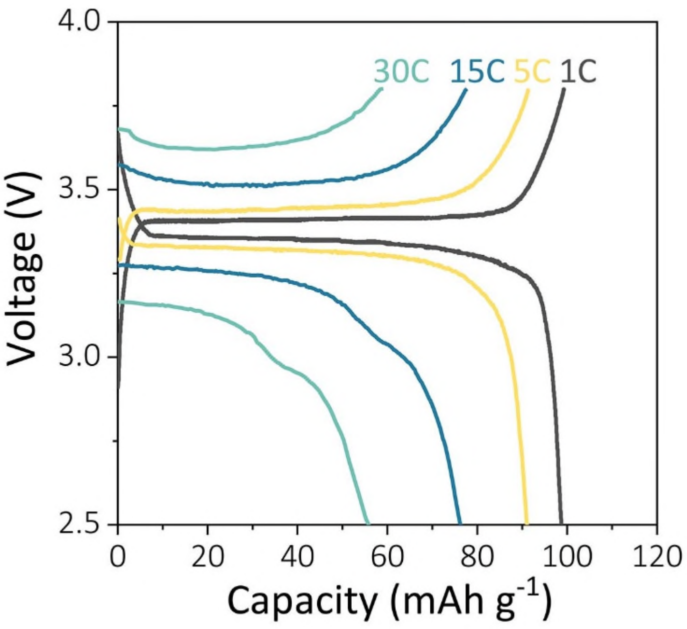

Supplementary Figure 28. Galvanostatic charge-discharge profiles of Na || NVP@C full cells with PP separator obtained at varying C-rate.

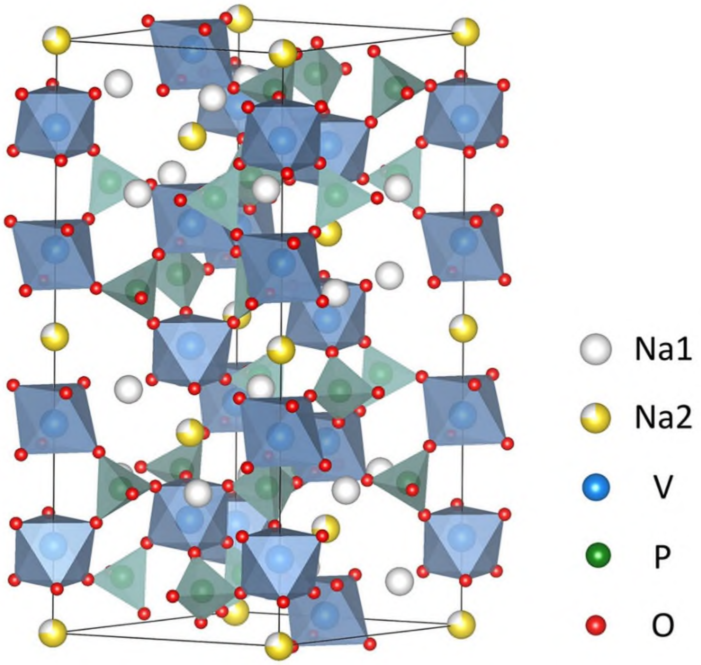

Supplementary Figure 29. Schematic diagram of the crystal structure of $Na_3V_2(PO_4)_3$ with two Na sites. It is worth noting that the $Na_3V_2(PO_4)_3$ possesses two types of Na ions with different oxygen environments: Na1 (sixfold coordination) and Na2 (eightfold coordination) sites. When Na^+ is extracted from $Na_3V_2(PO_4)_3$, two Na2-sited ions (2/3 occupancy) are easily extracted and the remaining one Na^+ is anchored in Na1 site (1 occupancy). At the subsequent Na^+ insertion process, the available sites for sodium accommodation become less and less along with the continuous sodiation, leading to a large polarization (especially under high current densities) at the end of the discharge[16-18].

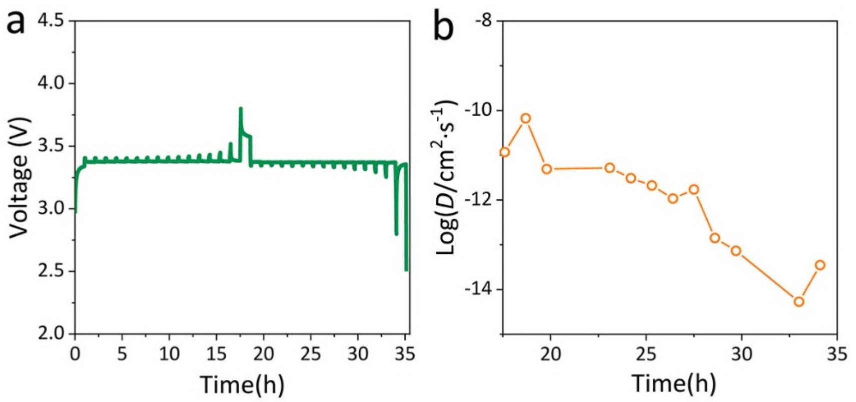

Supplementary Figure 30. (a) GITT profile of NVP@C during charge and discharge processes, (b) the D_{Na+} of NVP@C calculated from GITT curve during discharge process.

To measure the apparent sodium diffusion coefficient (D_{Na+}) in the charge/discharge process, the GITT technique was employed. Supplementary Fig. 30a exhibits the GITT curves of NVP@C as a function of time at 2.5-3.8 V. According to the Fick's second law of diffusion, the D_{Na+} of NVP@C is calculated by the following equation[9, 19-21].

$$D_{Na^+} = \frac{4}{\pi\tau}\left(\frac{m_B V_M}{M_B S}\right)^2 \left(\frac{\Delta E_S}{\Delta E_\tau}\right)^2 \quad (4)$$

Where τ is the duration time of the current pulse, m_B is the mass of electrode material, M_B is the molecular weight, V_M is the molar volume, S is the geometric area of electrode, ΔE_S is the difference between two consecutive stable voltages after relaxation, and ΔE_τ represents the transient voltage-change during a single titration step.

Clearly, the D_{Na+} of NVP@C is ranging from about 10^{-14} to 10^{-10} cm^2 s^{-1} during the discharge process, and decreases with the depth of discharge (Supplementary Fig. 30b). The reduced D_{Na+} could result in large polarization and induce the emergence of small platform close to the end of the discharge (specifically at high rates).

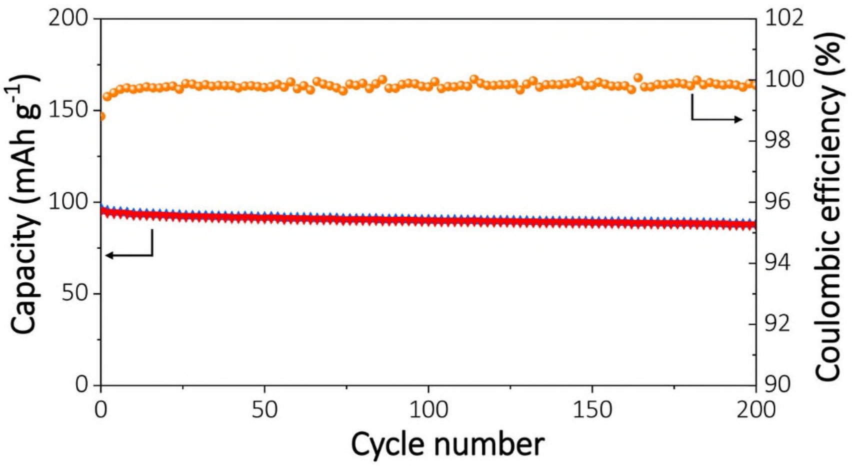

Supplementary Figure 31. Cycling stability of Na || NVP@C full cell with mPG-12@PP separator and reduced as-deposited Na anode of 5 mAh cm^{-2} obtained at 1 C.

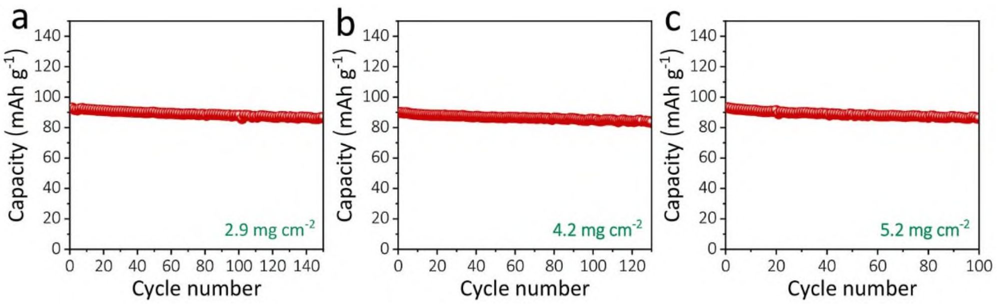

Supplementary Figure 32. Cycling stability of Na || NVP@C full cells with NVP@C loading of (a) 2.9, (b) 4.2 and (c) 5.2 mg cm^{-2} at 0.5 C, respectively. When the mass loading of NVP@C is increased to 3.2 mg cm^{-2}, corresponding Na || NVP@C full cell exhibits good cycling stability with 93.2% retention of the initial capacity over 150 cycles. Even at high NVP@C loading of 4.2 and 5.2 mg cm^{-2}, the cycling performance of Na || NVP@C full cells are still impressive, i.e., 92.8% for 120 cycles and 92.5% for 100 cycles at 0.5 C, respectively.

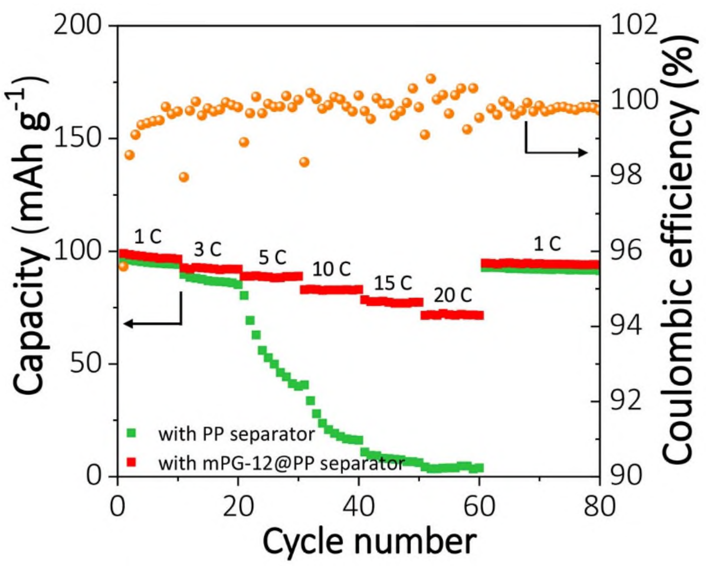

Supplementary Figure 33. Rate capability of Na || NVP@C full cells with mPG-12@PP and PP separator at different current density, reduced deposition of Na anode (5 mAh cm^{-2}), and relatively high loading of NVP@C (3.8 mg cm^{-2}).

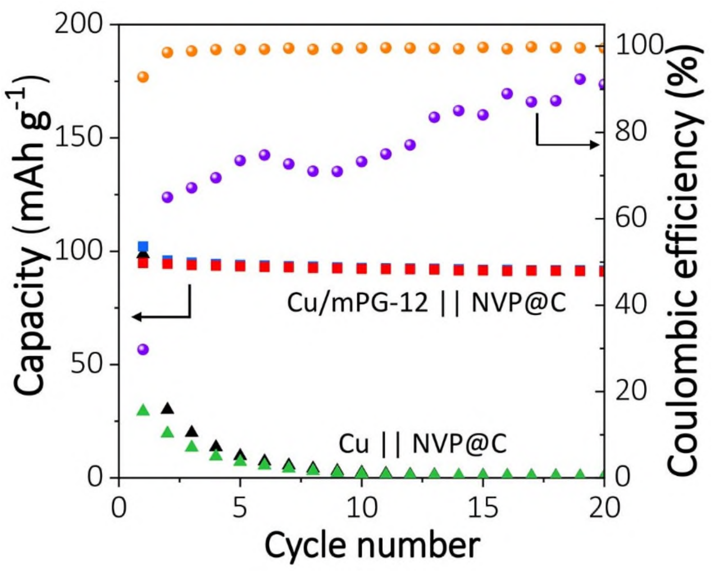

Supplementary Figure 34. Electrochemical performance of Cu/mPG-12 || NVP@C and Cu || NVP@C cells tested at 0.5 C.

When removing the metallic Na anode at initial cell fabrication of Na metal batteries, the safety hazards and the assembly cost will be greatly reduced, and the energy density can be remarkably improved[14, 22-29]. To this end, Cu || NVP@C cells with and without mPG-12 coated separators (defined as Cu/mPG-12 || NVP@C and Cu || NVP@C cells) were fabricated and tested by pairing NVP@C cathode with a bare Cu current collector on the anode side. With similar charge capacity in the first cycle, Cu/mPG-12 || NVP@C cell delivers an initial discharge capacity of 95 mAh g^{-1} and 96% capacity retention for 20 cycles, much higher than those of Cu || NVP@C cell (initial capacity of 29 mAh g^{-1} and 2% retention after 20 cycles). Also, the Cu/mPG-12 || NVP@C cell presents an increased average Coulombic efficiency of 99.1% compare to Cu || NVP@C (76.0%).

Supplementary Table 1. Structural parameters of mPG-7, mPG-12, mPG-22 and nPG obtained by N_2 adsorption and desorption measurements.

Sample	Surface area (m^2 g^{-1})	Pore volume (cm^3 g^{-1})	Pore size (nm)
mPG-7	144	0.23	6.8
mPG-12	157	0.26	12.2
mPG-22	114	0.30	18.6
nPG	54	~	~

Supplementary Table 2. **Coulombic efficiency and cycle life comparison of different Na metal anodes stabilized by various strategies in Na || Cu configuration.**

Strategies of Na anode	Current density (mA cm^{-2})	Areal capacity (mAh cm^{-2})	Coulombic efficiency	Refs.
Na@3D MXene-MF	5	5	99% for 100 cycles	30
Na@PVDF	1	1	99.91% for 1000 cycles	31
	2	1	99.48% for 1000 cycles	31
Na@N-doped graphene	0.5	1	99% for 800 cycles	32
	1	2	99% for 200 cycles	32
N, S- doped CNTs interlayer	1	1	99.82% for 400 cycles	33
Na@3D NVP	1	1	98% for 190 cycles	34
	1	2	96% for 76 cycles	34
Na@C@Sb	1	4	99.72% for 738 cycles	35
	2	4	99.74% for 880 cycles	35
Na@Sb_2MoO_6	5	4	95.2% for 500 cycles	36
Na@ 3D O-doped CNT	1	1	99.7% for 3000 cycles	37
	5	10	99.5% for 680 cycles	37
Na@Sn^{2+} pillared Ti_3C_2	4	3	99.1% for 300 cycles	38
	10	3	98.5% for 100 cycles	38
Na@O-doped CNTs	0.5	1	99.8% for 1000 cycles	39
	3	1	99.8% for 1000 cycles	39
$NaBF_4$/diglyme electrolyte	0.5	/	99.93% for 400 cycles	40
mPG-12@PP separator	0.5	0.5	99.8% for 550 cycles	This work
	4	4	99.9% for 450 cycles	
	8	8	99.7% for 350 cycles	

MF: melamine foam.

Supplementary Table 3. Cycle life comparison of different Na metal anodes stabilized by various strategies in Na || Na cell configuration.

Strategies of Na anode	Current density ($mA\ cm^{-2}$)	Areal capacity ($mAh\ cm^{-2}$)	Cycle time (h)	Refs.
Na@3D MXene-MF	10	10	720	30
Na@rGO	5	5	300	41
	1	1	600	41
Na@PVDF	1	1	1200	31
Na@N-doped graphene	1	1	1000	32
PhS_2Na_2 protection layer	1	1	800	42
Na@carbonized wood	1	1	500	43
N, S- doped CNTs interlayer	1	1	500	33
Na@3D NVP	1	1	300	34
Na@O-doped CNTs	1	1	250	39
Na@porous Cu	1	1	200	44
mPG-12@PP separator	1	1	2000	This work
	5	5	1700	
	10	10	1400	

PhS_2Na_2: sodium benzenedithiolate.

Supplementary Table 4. Rate performance comparison of Na metal anode in this work with the recently reported works using Na || Na cell configuration.

Strategies of Na anode	Current density (mA cm^{-2})	Areal capacity (mAh cm^{-2})	Refs.
Na@3D MXene-MF	20	20	30
Na@Hg alloy	8	8	45
Na@C@Sb	5	1	35
Na@Sb_2MoO_6	10	8	36
Na@ 3D O-doped CNT	10	2	37
Na@rGO aerogel	5	5	46
Na@Sn^{2+} pillared Ti_3C_2	8	3	38
Na@porous Cu	3	1	44
Na@3D carbon felt	5	2	47
mPG-12@PP separator	25	25	This work

References

1. Banhart F., Kotakoski J., Krasheninnikov A.V. Structural defects in graphene. *ACS Nano* **5**, 26-41 (2011).

2. Eckmann A., et al. Probing the nature of defects in graphene by Raman spectroscopy. *Nano Lett.* **12**, 3925-3930 (2012).

3. Eigler S., Dotzer C., Hirsch A. Visualization of defect densities in reduced graphene oxide. *Carbon* **50**, 3666-3673 (2012).

4. Liu W., et al. Pristine or highly defective? Understanding the role of graphene structure for stable lithium metal plating. *Adv. Energy Mater.* **9**, 1802918 (2019).

5. Moon I.K., Lee J., Ruoff R.S., Lee H. Reduced graphene oxide by chemical graphitization. *Nat. Commun.* **1**, 73 (2010).

6. Liu Y., et al. A polydopamine-modified reduced graphene oxide (RGO)/MOFs nanocomposite with fast rejection capacity for organic dye. *Chem. Eng. J.* **359**, 47-57 (2019).

7. Cui M., Ren S., Zhao H., Xue Q., Wang L. Polydopamine coated graphene oxide for anticorrosive reinforcement of water-borne epoxy coating. *Chem. Eng. J.* **335**, 255-266 (2018).

8. Yue X., Liu H., Liu P. Polymer grafted on carbon nanotubes as a flexible cathode for aqueous zinc ion batteries. *Chem. Commun.* **55**, 1647-1650 (2019).

9. Zhou Y., et al. A high-temperature Na-ion battery: Boosting the rate capability and cycle life by structure engineering. *Small* **16**, e1906669 (2020).

10. Bredar A.R.C., Chown A.L., Burton A.R., Farnum B.H. Electrochemical impedance

spectroscopy of metal oxide electrodes for energy applications. *ACS Appl. Energy Mater.* **3**, 66-98 (2020).

11. Liu W., Lin D., Pei A., Cui Y. Stabilizing lithium metal anodes by uniform Li-ion flux distribution in nanochannel confinement. *J. Am. Chem. Soc.* **138**, 15443-15450 (2016).
12. Zhang H., et al. Lithiophilic-lithiophobic gradient interfacial layer for a highly stable lithium metal anode. *Nat. Commun.* **9**, 3729 (2018).
13. Li T.J., et al. Superior sodium metal anodes enabled by sodiophilic carbonized coconut framework with 3D tubular structure. *Adv. Energy Mater.* **11**, 2003699 (2021).
14. Cohn A.P., Muralidharan N., Carter R., Share K., Pint C.L. Anode-free sodium battery through in situ plating of sodium metal. *Nano Lett.* **17**, 1296-1301 (2017).
15. Yang C., et al. Ultrafine silver nanoparticles for seeded lithium deposition toward stable lithium metal anode. *Adv. Mater.* **29**, 1702714 (2017).
16. Zhang X., et al. $Na_3V_2(PO_4)_3$: An advanced cathode for sodium-ion batteries. *Nanoscale* **11**, 2556-2576 (2019).
17. Chen S., et al. Challenges and perspectives for NASICON-type electrode materials for advanced sodium-ion batteries. *Adv. Mater.* **29**, 1700431 (2017).
18. Jian Z., et al. Atomic structure and kinetics of NASICON $Na_xV_2(PO_4)_3$ cathode for sodium-ion batteries. *Adv. Funct. Mater.* **24**, 4265-4272 (2014).
19. Zhang W., et al. Full activation of Mn^{4+}/Mn^{3+} redox in $Na_4MnCr(PO_4)_3$ as a high-voltage and high-rate cathode material for sodium-ion batteries. *Small* **16**, e2001524 (2020).
20. Rui X., et al. A low-temperature sodium-ion full battery: Superb kinetics and cycling stability. *Adv. Funct. Mater.* **31**, 2009458 (2021).

21. Zhang J., et al. A novel NASICON-type $Na_4MnCr(PO_4)_3$ demonstrating the energy density record of phosphate cathodes for sodium-ion batteries. *Adv. Mater.* **32**, e1906348 (2020).

22. Louli A.J., et al. Diagnosing and correcting anode-free cell failure via electrolyte and morphological analysis. *Nat. Energy* **5**, 693-702 (2020).

23. Cohn A.P., et al. Rethinking sodium-ion anodes as nucleation layers for anode-free batteries. *J. Mater. Chem. A* **6**, 23875-23884 (2018).

24. Nanda S., Gupta A., Manthiram A. Anode-free full cells: A pathway to high-energy density lithium-metal batteries. *Adv. Energy Mater.* **11**, 2000804 (2020).

25. Weber R., et al. Long cycle life and dendrite-free lithium morphology in anode-free lithium pouch cells enabled by a dual-salt liquid electrolyte. *Nat. Energy* **4**, 683-689 (2019).

26. Martin C., Genovese M., Louli A.J., Weber R., Dahn J.R. Cycling lithium metal on graphite to form hybrid lithium-ion/lithium metal cells. *Joule* **4**, 1296-1310 (2020).

27. Alvarado J., et al. Bisalt ether electrolytes: A pathway towards lithium metal batteries with Ni-rich cathodes. *Energy Environ. Sci.* **12**, 780-794 (2019).

28. Zhang J.-G. Anode-less. *Nat. Energy* **4**, 637-638 (2019).

29. Tang S., et al. A room-temperature sodium metal anode enabled by a sodiophilic layer. *Nano Energy* **48**, 101-106 (2018).

30. Shi H., et al. 3D flexible, conductive, and recyclable $Ti_3C_2T_x$ MXene-melamine foam for high-areal-capacity and long-life time alkali-metal anode. *ACS Nano* **14**, 8678-8688 (2020).

31. Hou Z., et al. Poly(vinylidene difluoride) coating on Cu current collector for high-performance Na metal anode. *Energy Storage Mater.* **24**, 588-593 (2020).

32. Bao C., et al. Sodiophilic decoration of a three-dimensional conductive scaffold toward a stable Na metal anode. *ACS Sustainable Chem. Eng.* **8**, 5452-5463 (2020).

33. Sun B., et al. Dendrite-free sodium-metal anodes for high-energy sodium-metal batteries. *Adv. Mater.* **30**, 1801334 (2018).

34. Guo M., et al. Three dimensional frameworks of super ionic conductor for thermodynamically and dynamically favorable sodium metal anode. *Nano Energy* **70**, 104479 (2020).

35. Wang G., et al. Core-shell C@Sb nanoparticles as a nucleation layer for high-performance sodium metal anodes. *Nano Lett.* **20**, 4464-4471 (2020).

36. Lu X., et al. Enabling high-performance sodium metal anodes via a sodiophilic structure constructed by hierarchical Sb_2MoO_6 microspheres. *Nano Energy* **69**, 104446 (2020).

37. Ye L., et al. A sodiophilic interphase-mediated, dendrite-free anode with ultrahigh specific capacity for sodium-metal batteries. *Angew. Chem. Int. Ed.* **58**, 17054-17060 (2019).

38. Luo J., et al. Pillared MXene with ultralarge interlayer spacing as a stable matrix for high performance sodium metal anodes. *Adv. Funct. Mater.* **29**, 1805946 (2019).

39. Chu C., et al. Uniform nucleation of sodium in 3D carbon nanotube framework via oxygen doping for long-life and efficient Na metal anodes. *Energy Storage Mater.* **23**, 137-143 (2019).

40. Wang S., et al. Stable sodium metal batteries via manipulation of electrolyte solvation structure. *Small Methods* **4**, 1900856 (2020).

41. Wang A., et al. Processable and moldable sodium-metal anodes. *Angew. Chem. Int. Ed.* **56**, 11921-11926 (2017).

42. Zhu M., et al. Dendrite-free sodium metal anodes enabled by a sodium benzenedithiolate-rich protection layer. *Angew. Chem. Int. Ed.* **59**, 6596-6600 (2020).

43. Luo W., et al. Encapsulation of metallic Na in an electrically conductive host with porous channels as a highly stable Na metal anode. *Nano Lett.* **17**, 3792-3797 (2017).

44. Wang C., Wang H., Matios E., Hu X., Li W. A chemically engineered porous copper matrix with cylindrical core-shell skeleton as a stable host for metallic sodium anodes. *Adv. Funct. Mater.* **28**, 1802282 (2018).

45. Zhang Q., et al. A thermodynamically stable quasi-liquid interface for dendrite-free sodium metal anodes. *J. Mater. Chem. A* **8**, 6822-6827 (2020).

46. Wu F., et al. Reduced graphene oxide aerogel as stable host for dendrite-free sodium metal anode. *Energy Storage Mater.* **22**, 376-383 (2019).

47. Chi S.-S., Qi X.-G., Hu Y.-S., Fan L.-Z. 3D flexible carbon felt host for highly stable sodium metal anodes. *Adv. Energy Mater.* **8**, 1702764 (2018).
